# Transient Seizure Clusters and Epileptiform Activity Following Widespread Bilateral Hippocampal Interneuron Ablation

**DOI:** 10.1523/ENEURO.0317-23.2024

**Published:** 2024-04-16

**Authors:** Mary R. Dusing, Candi L. LaSarge, Austin W. Drake, Grace C. Westerkamp, Carlie McCoy, Shelby M. Hetzer, Kimberly L. Kraus, Ernest V. Pedapati, Steve C. Danzer

**Affiliations:** ^1^Department of Anesthesia, Cincinnati Children’s Hospital Medical Center, Cincinnati, Ohio 45229-3039; ^2^Neuroscience Graduate Program, University of Cincinnati, Cincinnati, Ohio 45229-3039; ^3^Medical Scientist Training Program, University of Cincinnati College of Medicine, Cincinnati, Ohio 45229-3039; ^4^Division of Child Psychiatry, Cincinnati Children’s Hospital Medical Center, Cincinnati, Ohio 45229-3039; ^5^Division of Neurosurgery, Cincinnati Children’s Hospital Medical Center, Cincinnati, Ohio 45229-3039; ^6^Department of Anesthesiology, University of Cincinnati College of Medicine, Cincinnati, Ohio 45229-3039

**Keywords:** diphtheria toxin, DREADDs, epileptogenesis, neuropeptide Y, parvalbumin, Vgat

## Abstract

Interneuron loss is a prominent feature of temporal lobe epilepsy in both animals and humans and is hypothesized to be critical for epileptogenesis. As loss occurs concurrently with numerous other potentially proepileptogenic changes, however, the impact of interneuron loss in isolation remains unclear. For the present study, we developed an intersectional genetic approach to induce bilateral diphtheria toxin-mediated deletion of Vgat-expressing interneurons from dorsal and ventral hippocampus. In a separate group of mice, the same population was targeted for transient neuronal silencing with DREADDs. Interneuron ablation produced dramatic seizure clusters and persistent epileptiform activity. Surprisingly, after 1 week seizure activity declined precipitously and persistent epileptiform activity disappeared. Occasional seizures (≈1/day) persisted to the end of the experiment at 4 weeks. In contrast to the dramatic impact of interneuron ablation, transient silencing produced large numbers of interictal spikes, a significant but modest increase in seizure occurrence and changes in EEG frequency band power. Taken together, findings suggest that the hippocampus regains relative homeostasis—with occasional breakthrough seizures—in the face of an extensive and abrupt loss of interneurons.

## Significance Statement

Interneuron loss is hypothesized to play a critical role in epileptogenesis; however, the co-occurrence of interneuron loss with other potentially epileptogenic changes has made assigning causal relationships challenging. Here, we utilized an intersectional genetic approach to delete hippocampal interneurons. Treatment produced robust—but transient—seizure clusters in the animals followed by a relative recovery with infrequent seizures. Findings support a critical role for interneuron loss in epileptogenesis but also imply the existence of mechanisms that can rapidly restore excitatory/inhibitory balance in the brain.

## Introduction

The cause of epilepsy is often attributed to an imbalance between excitation and inhibition in the brain. While oversimplistic, the general concept is supported by a large body of data, particularly with regard to impaired inhibition. Many genetic causes of human epilepsy involve genes regulating GABAergic inhibitory neuron development or function (Feng et al., 2022; Maillard et al., 2022), many antiseizure medications enhance GABA signaling (Löscher and Klein, 2021), and acquired epilepsy is consistently associated with GABA neuron dysfunction and loss in both patients (Williamson et al., 1999; Tóth et al., 2010; DeLanerolle, 2012; Thom, 2014) and animal models (Ribak et al., 1979; Sloviter, 1991; Houser, 2014; Wyeth et al., 2020; LaSarge et al., 2021). Moreover, acute pharmacological blockade of GABA signaling has served as a tried-and-true means of generating seizures for decades using both in vivo and in vitro experimental preparations. Conversely, conditions under which GABA can depolarize neurons, and the ability of interneurons to promote seizures by synchronizing excitatory neuron activity, have been explored as ictogenic mechanisms (Chang et al., 2018; Dossi and Huberfeld, 2023; Trevelyan et al., 2023; van van Hugte et al., 2023). Such observations have led investigators to hypothesize that interneuron dysfunction or loss is an essential, if not the key, driver of epileptogenesis (Martin and Sloviter, 2001; Chun et al., 2019).

A persistent challenge for elucidating the impact of interneuron loss in temporal lobe epilepsy, however, is that loss never occurs in isolation. Rather, interneuron loss co-occurs with a host of other brain changes which might also be epileptogenic. Favoring a driving role in acquired temporal lobe epilepsy models, interneuron loss typically occurs prior to or concurrent with the onset of spontaneous seizures (Olney et al., 1986; Niquet et al., 2012) and correlates with seizure frequency (Buckmaster et al., 2017). On the other hand, while interneuron loss occurs within days in status epilepticus models, seizure frequency increases for weeks to months after the insult (Williams et al., 2009; Dudek and Staley, 2012; Van Nieuwenhuyse et al., 2015; Hosford et al., 2017), implying that additional neuroplastic changes occurring during this timeframe are critical for driving epilepsy progression. The relative importance of interneuron loss in temporal lobe epilepsy, thus, remains unclear.

A variety of approaches have been developed to manipulate interneurons. Deletion of genes regulating interneuron development can reduce interneuron number and produce seizures (Cobos et al., 2005; Bolneo et al., 2022); however, such approaches can also disrupt brain development more broadly (Wang et al., 2010) and poorly replicate the time course of interneuron loss in acquired epilepsies, which occurs quickly following an epileptogenic event (e.g., status epilepticus, traumatic brain injury). The development of intersectional genetic approaches to manipulate specific neuron types, on the other hand, provides a means to model the acute loss of interneurons in adult animals. Using such approaches, several investigators have demonstrated that ablation or silencing of interneuron subtypes can lead to seizures (Drexel et al., 2017, 2022; Spampanato and Dudek, 2017) and epileptiform activity (Panthi and Leitch, 2019). Interestingly, however, while the investigators found that interneuron ablation did cause seizures, seizure incidence was modest, and animals often went into remission within a few weeks. These prior studies utilized restricted unilateral ablations in CA1 or subiculum or targeted specific interneuron subtypes. To determine whether more expansive interneuron loss would cause a more severe phenotype, for the present study we conducted bilateral ablation of vesicular GABA transporter (Vgat)-expressing interneurons in dorsal and ventral hippocampus, allowing us to target a majority of interneuron subtypes. A parallel cell-silencing strategy was used to compare the effects of transient verses permanent interneuron disruption.

## Materials and Methods

### Animals

*Slc32a1*^tm1.1(flpo)Hze^/J (Vgat-FlpO) mice were obtained from the Jackson Laboratory on a C57BL/6J background. Mice were then backcrossed onto a C57BL/6N background for six generations. Experiments were conducted on 10.5–13-week-old male and female mice randomized to treatment group (Table 1). All animal procedures were conducted in accord with the NIH guidelines and with approval of CCHMC’s IACUC committee.

**Table 1.** Experimental groups

Exp. #	Exp.	Readout	Group	Genotype	AAV	Treatment	*N*
1	Vgat Ablation	EEG	Control	Vgat-FlpO −/−	AAV9-CAG-frt-DTr	Diphtheria toxin	7 (3M, 4F)
			Control	Vgat-FlpO +/−	AAV9-CAG-frt-DTr	Saline	5 (3M, 2F)
			Ablation	Vgat-FlpO +/−	AAV9-CAG-frt-DTr	Diphtheria toxin	10 (6M, 4F)
2	DT control	EEG	Control	Vgat-FlpO +/−	AAV9-frt-mCherry	Diphtheria toxin	4 (2M, 2F)
3	Vgat Silencing	EEG	Control	Vgat-FlpO −/−	AAV9-CAG-frt- hM4D_i_/HA	Saline, CNO, Saline, CNO	6 (5M, 1F)
			Silencing	Vgat-FlpO +/−	AAV9-CAG-frt- hM4D_i_/HA	Saline, CNO, Saline, CNO	4 (3M, 1F)

### Plasmid constructs

**Figure 1.**
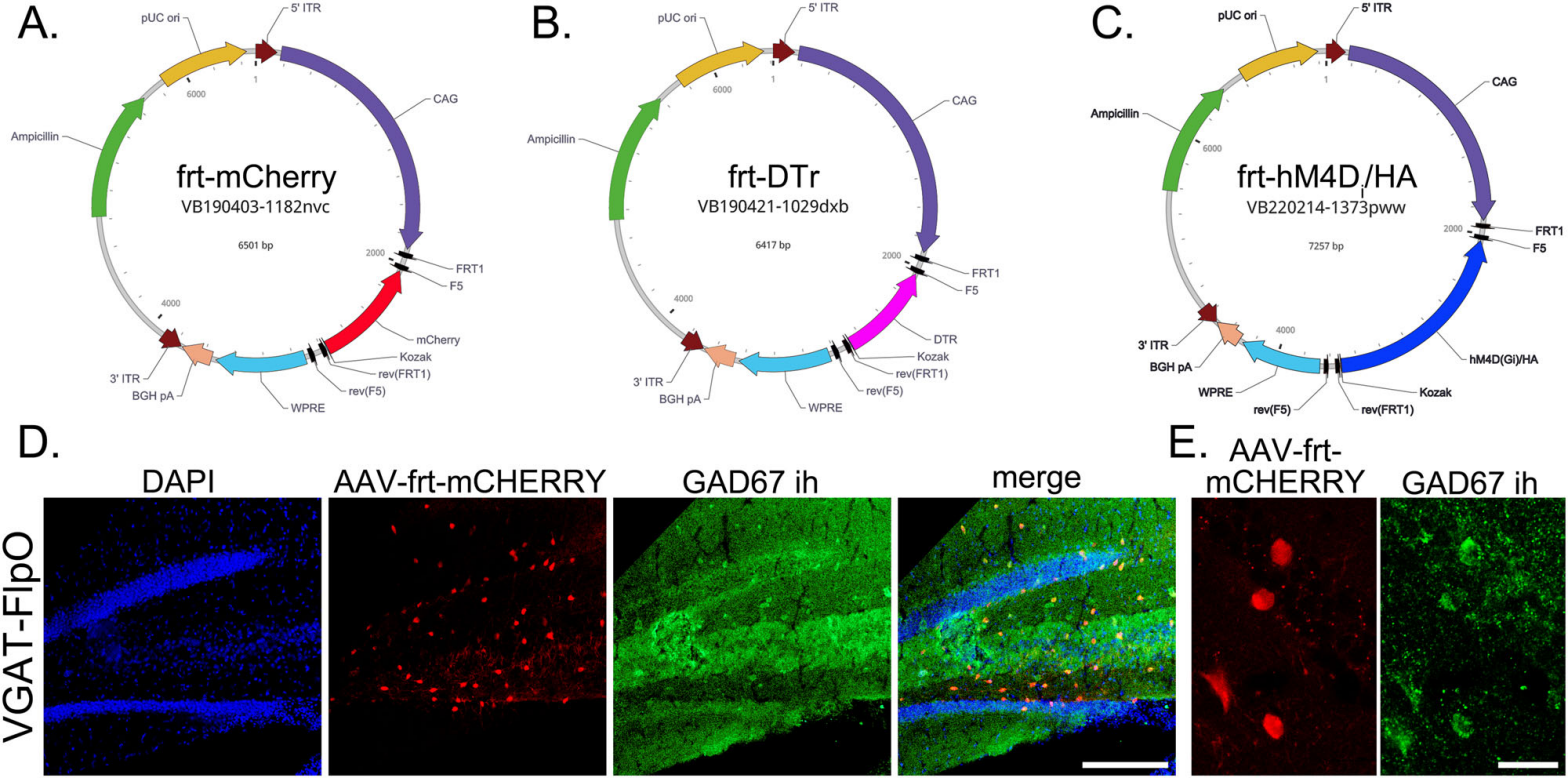
***A***, Plasmid map for the FlpO-dependent (frt) mCherry reporter. ***B***, Plasmid map for the frt-DTr expression vector. ***C***, Plasmid map for the frt-hM4D_i_/HA (DREADD) expression vector. ***D***, The specificity of Vgat-FlpO mice was tested by injecting animals with AAV9-frt-mCherry (red), immunostaining hippocampi with the GABAergic marker Gad67 (green), and counterstaining with DAPI (blue). mCherry and Gad67 are extensively colocalized in the dentate gyrus, with little to no labeling of excitatory granule cells. Scale bar, 200 µm. ***E***, High resolution images show colocalization of mCherry in Gad67-immunoreactive hilar interneurons. Scale bar, 25 µm.

Plasmids (Fig. 1) contained the CMV early enhancer fused to modified chicken beta-actin (CAG) promoter sequence. An inverted mCherry reporter (VB190403-1182nvc, pAAV[Exp]-CAG > FRT1-F5-rev[mCherry]-rev[FRT1]-rev[F5]:WPRE), diphtheria toxin receptor (VB190421-1029dxb, pAAV[Exp]-CAG > FRT1-F5-rev[DTr]-rev[FRT1]-rev[F5]:WPRE), or hM4D_i_/HA (VB220214-1373pww, pAAV[Exp]-CAG > FRT1-F5-rev[hM4D-Gi/HA]-rev[FRT1]-rev[F5]:WPRE) flanked by a pair of flippase recombination (FRT1/F5) sites was cloned 3′ to the CAG promoter followed by the woodchuck hepatitis virus posttranscriptional regulatory element (WPRE) and the bovine growth hormone polyadenylation signal (BGH pA). Plasmids were produced and packaged into the AAV9 serotype by VectorBuilder.

### Virus injection and EEG electrode implantation

Virus was infused through two sets of bilateral holes drilled through the skull at the following coordinates (relative to bregma, in mm): dorsal: (AP) −2.3, (ML) ±1.3, depth −2.5 from skull surface, and ventral: (AP) −3.0, (ML) ±2.75, depth −2.75. Virus was infused into the hippocampus with a Hamilton syringe at a flow rate of 100 nl/min over 5 min. A total of 500 nl of virus was injected per site for total viral infusion of 2 × 10^8^ viral genomes per animal. Animals were injected with either AAV9-CAG-frt-mCherry, AAV9-CAG-frt-DTr, or AAV9-CAG-frt-hM4D_i_/HA to express mCherry, the diphtheria toxin receptor (DTr), or the chemogenetic (DREADD) silencing molecule hM4D_i_, respectively. Viral constructs were diluted to the same titers and injected at identical coordinates and volumes to facilitate comparison between ablation and silencing experiments.

For experiments requiring 24/7 video-EEG monitoring, two-lead wireless transmitters (TA11ETA-F10; Data Sciences International) were implanted immediately following virus injection in accord with previous studies (Rowley et al., 2017; Tiwari et al., 2020). Cortical electrode leads were placed just over the dura using the holes drilled for virus injection into the dorsal hippocampus. Electrode wires feed into the transmitter, which was placed subcutaneously under the back of each animal. Cortical leads were secured with dental cement and the incision closed with surgical sutures. Neosporin (per gram ointment: 500 units bacitracin, 3.5 mg neomycin, 10,000 units polymyxin B) was swabbed around the incision. Carprofen (5 mg/kg, i.p.) was given immediately after the surgery and again 24 h later. Hydration was given as needed, to maintain pretreatment body weight, with subcutaneous injections of sterile saline. All animals were individually housed after surgery.

### Experiment 1: diphtheria toxin treatment and EEG monitoring of Vgat ablation mice

EEG-implanted mice injected with AAV9-CAG-frt-DTr were allowed to recover for 2 weeks after surgery (Table 1, Experiment 1), followed by a week of baseline 24/7 video-EEG monitoring. Beginning in the fourth week after virus injections, mice were treated with diphtheria toxin or saline. Diphtheria toxin (List Biological Laboratories, #150) was prepared in sterile normal saline at 0.1 mg/ml and stored frozen in aliquots at −80°C. Diphtheria toxin was diluted to a concentration of 5 ng/µl the day of use in normal saline. Mice were injected intraperitoneally at 20 µg/kg with diphtheria toxin once per day for 5 d. Video-EEG monitoring continued through treatment and for an additional 2–4 weeks after treatment.

### Experiment 2: diphtheria toxin treatment and EEG monitoring of Vgat-FlpO–positive, DTr-negative mice

To confirm that Vgat-FlpO–expressing mice were not uniquely sensitive to nonspecific effects of diphtheria toxin, four Vgat-FlpO+/− animals were injected with AAV9-frt-mCherry and implanted with EEG electrodes (Table 1, Experiment 2). Animals were EEG recorded and treated with diphtheria toxin as described for other groups. We continued 24/7 video-EEG monitoring for a minimum of 2 weeks after toxin treatment, after which brains were collected for histological studies.

### Experiment 3: hM4Di-mediated interneuron silencing and EEG monitoring

EEG-implanted mice injected with AAV9-CAG-frt-hM4D_i_/HA were allowed to recover for 3 weeks following surgery (Table 1, Experiment 3). Beginning in the fourth week after virus injections, mice began a crossover design study with an initial saline control treatment followed 2 d later by the first treatment with the hM4D_i_ ligand clozapine *N*-oxide (CNO, 10 mg/kg in normal saline, i.p.). Five days later the second saline treatment was given, followed by a second CNO treatment 2 d later. Saline and CNO injections were given between noon and 2 P.M. Brains were collected 1 week after treatment for histological studies.

### EEG analysis

EEG was collected at a sampling rate of 1,000 Hz. EEG data were scored by an investigator unaware of treatment group using NeuroScore software (DSI). Seizures were defined as events characterized by an abrupt increase in EEG amplitude (>2× baseline), a duration >10 s, and spikes that change in amplitude, frequency, or waveform shape during the event. Evolution of spike amplitude, frequency, or waveform is used to discriminate seizure events from spike runs, which can meet other seizure criteria but exhibit no change in spike parameters throughout the event. Persistent epileptiform activity was defined as periods of continuous spikes or polyspikes, spike wave discharges, and burst suppression which continued for >300 s. This included the occurrence of multiple electrographic seizures and repeated brief (<10 s) seizure-like events without recovery between episodes. Video for each seizure event was reviewed to assign a behavioral seizure score using a modified Racine scale (Racine, 1972), as follows: Class 1, freezing; Class 2, orofacial automatisms; class 3, unilateral forelimb clonus; Class 4, bilateral forelimb clonus; Class 5, rearing and falling; Class 6, wild running and bouncing “popcorn-like” seizures; Class 7, tonic extension and rigidity; and Class 8, continuous epileptiform activity. Following primary screening by the first investigator, EEG recordings were reviewed by a second investigator. Any discrepancies in scoring were resolved by the senior investigator (SCD).

For Vgat interneuron silencing experiments with hM4D_i_, the 3 h period immediately following CNO or saline treatment was screened using NeuroScore's automated spike detector. Detector settings were optimized to capture all interictal spikes (ISs) with high sensitivity, and then EEG files were manually reviewed for specificity and to remove artifacts miss-identified as IS. EEG data for the 3 h period following treatment was also used for EEG power calculations. Data were exported in EDF format and imported into MATLAB (version 2021b, The MathWorks) running EEGLAB SET v2022.0 with custom scripts (https://github.com/cincibrainlab/vhtp/blob/main/eeg_htpCalcRestPower.m). Files were then visually inspected for movement artifact contamination by a trained assistant blind to group. No more than 10% of any recording was rejected due to movement artifact (highest percentage of rejected data was 8.3%). For further analysis, EEG signals were processed by adding a 0.1 Hz high-pass filter, and continuous data files were segmented into 2 s epochs. Epochs underwent fast Fourier transforms using a Hanning window with 0.5 Hz bin resolution, yielding absolute power (μV/Hz^2^) data across a 1–100 Hz frequency range. Relative power was defined as the band-specific absolute power. To obtain relative power, the cumulative absolute power (*V*^2^/Hz) in each band was divided by the total power across all bands and averaged across the available trials. Frequency bands were defined as follows: delta, 0.5–4 Hz; theta, 4–8 Hz; alpha, 9–12 Hz; beta, 16–30 Hz; and gamma, 30–55 Hz.

### Tissue preparation

Mice were overdosed with pentobarbital (100 mg/kg) and perfused with phosphate-buffered saline (0.1 M PBS) +1 U/ml heparin, followed by 2.5% paraformaldehyde with 4% sucrose in 0.1 M PBS, pH 7.4. Brains were postfixed overnight, cryoprotected, frozen, and stored at −80°C. Coronal sections were cut at 40 µm onto gelatin-coated slides and stored at −80°C.

### Immunostaining

Brain sections from study animals were immunostained using the following antibodies: DTr (1:500 goat anti-HB EGF, #AF259SP, R&D Systems and 1:750 donkey anti-goat Alexa Fluor 568, #A11057, Thermo Fisher Scientific), GAD1/GAD67 (1:500 chicken anti-GAD1/GAD67, #198006, Synaptic Systems and 1:750 goat anti-chicken Alexa Fluor 488, #A11039, Thermo Fisher Scientific), somatostatin (1:500 rabbit anti-somatostatin, #PA5-82678, Invitrogen and 1:750 donkey anti-rabbit Alexa Fluor 647, #A-31573, Invitrogen), parvalbumin (1:1,000 guinea pig anti-parvalbumin, #195004, Synaptic Systems and 1:750 donkey anti-guinea pig DyLight 405, #706475148 Jackson ImmunoResearch or Alexa Fluor 647, #706605148, Jackson ImmunoResearch), neuropeptide Y (1:300 rabbit anti-neuropeptide Y, #N9528, Sigma-Aldrich and 1:750 donkey anti-rabbit Alexa Fluor 488, #R37118, Thermo Fisher Scientific or 1:750 goat anti-rabbit Alexa Fluor 488, #A11008, Thermo Fisher Scientific), hemagglutinin (1:500 rat anti-HA, #11867423001, Roche and 1:750 goat anti-rat Alexa Fluor 568, #A11077, Thermo Fisher Scientific), and gephyrin (1:1,000 chicken anti-gephyrin, #147009, Synaptic Systems and 1:750 donkey anti-chicken Alexa Fluor 488, #A78948, Invitrogen). Counterstains were Neurotrace Blue (1:750, #N21479, Thermo Fisher Scientific) or ProLong Glass mounting media containing nuclear blue, #P36983, Thermo Fisher Scientific).

### Neuroanatomical analyses

To characterize the pattern of DTr expression following hippocampal injection of AAV9-CAG-frt-DTr into Vgat-FlpO mice, serial sections through the rostral–caudal extent of the brain (10–15 sections per mouse spanning bregma 2.10 to −3.80) were examined from five animals treated with saline and immunostained for DTr. Staining by brain region was scored on a semiquantitative 0–3 scale (3, DTr present in >75% of a region; 2, ≈50% labeled; 1, <25%; 0, no labeling).

Brain sections immunostained for parvalbumin and somatostatin were used to determine the density of these immunolabeled cells within the hippocampus. All images for each interneuron marker were collected with identical microscope settings (Nikon Apo LWD 20× objective; NA = 0.95; 2 µm step through 20 µm *z*-depth). Parvalbumin and somatostatin neurons were counted in the left and right hemispheres of two brain sections (one dorsal, one ventral) per mouse, giving a total of four hippocampi per mouse. Counts were conducted using Nikon NIS-Elements software (RRID:SCR_014329). Values were converted to density ([cell number / (*z*-depth × hippocampal area)] × 1,000,000) and measures from the left and right hemisphere at each level were averaged for statistical analyses.

Parvalbumin interneurons support large axonal arbors, which can be revealed by parvalbumin immunostaining. As an additional measure of parvalbumin loss, we quantified reductions in parvalbumin immunoreactivity in different hippocampal subfields. Signal consists primarily of these large axonal arbors. Confocal images were collected from brain sections covering the dorsal–ventral extent of the hippocampus (typically 6–7 sections per mouse at 500 µm intervals). All images were collected with identical microscope settings (Nikon A1+; Plan Apo 4× objective; NA = 0.2). For each section, the entire hippocampus was imaged in both hemispheres. NIS-Elements software was used to draw regions of interest (ROIs) around the entire hippocampus as well as the dentate gyrus, CA3 region, and CA1 region, including all sublayers. An ROI from the corpus callosum immediately above the midpoint of CA1 was also created for each sample and hemisphere. Average intensity values for each ROI were collected. Intensity values were normalized to corpus callosum to control for differences in background staining [(ROI-ipsilateral corpus callosum) / ipsilateral corpus callosum] × 100.

To quantify granule cell perisomatic inhibitory inputs, we examined the brain sections from control and Vgat ablation mice immunostained for parvalbumin and gephyrin. Confocal images were collected from the midpoint of the upper blade of the dentate granule cell layer (two images per mouse from left and right hemispheres; Nikon Ti2; Plan Apo 100× oil objective; NA = 1.45; 0.2 µm step; resolution 0.09 µm/pixel). Perisomatic inputs were quantified using NIS-Elements software to manually measure soma perimeter and mark puncta. Only granule cells located within 0–2 soma diameters of the granule cell–molecular layer border were scored. Somas were also excluded if >50% of the soma perimeter was immediately adjacent to another granule cell. Five cells per hemisphere were scored (10 cells/mouse). To avoid bias, somas were selected for quantification by viewing only the Neurotrace Blue channel, beginning at the top of the image and selecting the first five cells meeting inclusion criteria. Puncta were quantified from a single optical section through the midpoint of selected granule cell somas. To quantify puncta, a line was first drawn around the soma using the Neurotrace Blue channel to capture the perimeter. Next, all parvalbumin and gephyrin immunoreactive puncta touching this line in the selected optical section were counted. Total gephyrin and parvalbumin puncta were normalized to cell perimeter. Parvalbumin puncta that touched gephyrin puncta were marked as “apposed” and converted to a percentile ([apposed puncta / total gephyrin puncta] × 100). The 10 soma measurements per animal were averaged for statistical analysis.

### Data analysis and statistics

All data collection was conducted by investigators blind to animal genotype and treatment group. Statistical analyses were conducted using Sigma Plot (version 14.5) or GraphPad Prism (version 9.5). No significant effects of animal sex were found (data not shown), so males and females were pooled for analysis. Control groups did not differ significantly from each other either (data not shown), so these were also pooled. Datasets were screened for normality (Shapiro–Wilk) and equal variance (Brown–Forsythe). Parametric tests were used for data that met assumptions of normality and equal variance. For data that failed either test, nonparametric alternate tests were used when available. Vgat-silencing experiments (seizure frequency, spike frequency, and EEG power) were analyzed by two-way repeated measures ANOVA. This test lacks a clear nonparametric alternative, so rank, square root, or ln transformations were used when necessary to normalize the Vgat-silencing datasets and/or achieve equal variance. Seizure frequency data from Vgat ablation experiments could not be normalized and were thus analyzed using the nonparametric Mann–Whitney rank sum test (RST) with Bonferroni’s corrections for multiple comparisons. Specific tests used for each comparison are noted in the results. Alpha was set at 0.05 except as noted for Bonferroni’s corrections.

### Figure preparation

Figures were prepared using Adobe Photoshop (CS5 extended, version 12.0) and GraphPad Prism (version 9.5.0). Confocal images meant for comparison were collected with identical microscope settings and received identical adjustments to brightness and contrast to optimize cellular detail.

## Results

### Intersectional genetic targeting of Vgat-expressing interneurons

Mice expressing Flp recombinase (FlpO) inserted in the translational STOP codon of the vesicular inhibitory amino acid transporter (Vgat) gene Slc32a1 were used to selectively target GABAergic interneurons in the hippocampus. For the present study, animals received bilateral dorsal and ventral hippocampal injections of adeno-associated viral (AAV9) vectors containing frt-flanked inverted reporter genes (Fig. 1*A–C*). Inversion and expression of the reporter occurs only in cells expressing FlpO, in this case Vgat-expressing cells. To confirm the efficacy of the approach, animals were injected with AAV9-frt-mCherry followed by tissue collection and immunostaining with the interneuron marker GAD67. Cell labeling with mCherry was evident in the dentate hilus, where interneurons are concentrated (Fig. 1*D*). No evidence of widespread expression of mCherry was found among excitatory granule cells or hippocampal pyramidal cells (data not shown). Immunostaining confirmed selective expression of mCherry among GAD67-positive cells in the hilus (Fig. 1*E*).

### Ablation of hippocampal Vgat-expressing interneurons

**Figure 2.**
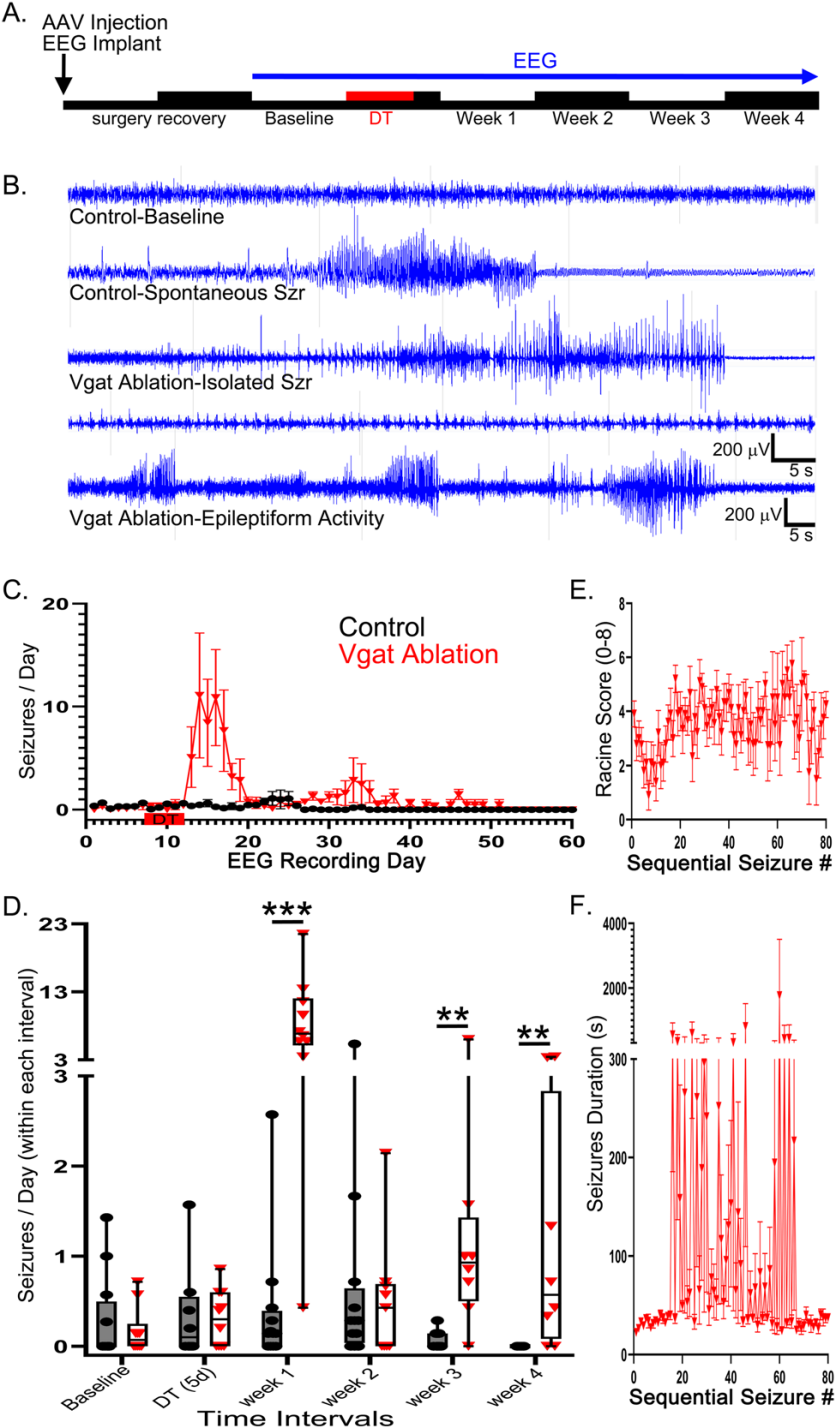
***A***, 24/7 video-EEG recording (blue line) began with a 1 week baseline, followed by 5 d of diphtheria toxin treatment (DT, red) and 4 weeks of post-treatment recording. ***B***, EEG traces show background activity and a spontaneous seizure (Szr) observed during the period of diphtheria toxin treatment in a control animal (top two traces). An isolated seizure from a Vgat ablation mouse is shown in the middle trace. The bottom two traces show activity scored as persistent epileptiform activity in Vgat ablation mice, including a burst suppression-like pattern, and rapid sequences of convulsive seizures without recovery between events. ***C***, Seizure number per recording day (mean ± SEM) showing seizure peaks in Vgat ablation mice in the first and third weeks after ablation. ***D***, Box and whisker (min to max) plots showing seizure number per day during the six recording windows. Note the break in the *y*-axis. **p < 0.01; ***p < 0.001. ***E***, Average behavioral seizure scores (mean ± SEM; modified Racine scale) by sequential seizure number for Vgat ablation mice. ***F***, Average seizure duration (mean ± SEM) by sequential seizure number for Vgat ablation mice. Long duration events between seizure numbers ≈15–65 reflect periods of persistent epileptiform activity. Note the break in the *y*-axis.

To determine whether ablation of Vgat-expressing neurons causes seizures, Vgat-FlpO+ mice received bilateral dorsal and ventral hippocampal injections of an FlpO-dependent DTr expression vector. The vector leads to DTr expression specifically in Vgat-expressing interneurons, which does not harm the cells unless the animals are treated with diphtheria toxin, in which case the expressing cells will be selectively eliminated. Similar approaches achieve effective and specific ablation of targeted cells (Saito et al., 2001; Buch et al., 2005; Hosford et al., 2016, 2017). Control Vgat-FlpO–negative animals received the same vector and diphtheria toxin treatment, while control Vgat-FlpO+ animals received the same vector and saline (Table 1, Experiment 1). Animals were video-EEG monitored for seven baseline days, followed by 5 d of diphtheria toxin or saline (control) treatment, and followed by 2–4 weeks of post-treatment monitoring (Fig. 2*A*). Example EEG traces for control and Vgat ablation mice are shown in Figure 2*B*, and seizure frequency by day is plotted in Figure 2*C*. For statistical comparisons, data were binned into six treatment windows, beginning with 1 week of baseline monitoring, the 5 d period of diphtheria toxin treatment, and Weeks 1–4 after toxin treatment (Fig. 2*D*).

### A subset of Vgat-FlpO mice have spontaneous seizures prior to treatment

Unexpectedly, baseline EEG monitoring revealed infrequent seizures in all groups (including controls) even after the 2 week surgery recovery period. We have only observed rare seizures among other mouse lines following surgery, even among concurrent studies with surgeries conducted by the same investigator (our unpublished observations). Although the cause of the baseline seizures is not known, it is possible that the Vgat-FlpO line has developed an epilepsy susceptibility mutation or that the creation of the line itself has disrupted GABAergic signaling. Consistent with the latter interpretation, the Vgat-Cre line has been reported to exhibit enhanced kindling epileptogenesis (Straub et al., 2020). Additional studies would be needed to elucidate the mechanisms underlying spontaneous seizures in the Vgat-FlpO mice; nonetheless, the observation highlights the importance of using littermate controls, as was done here.

Among DTr-expressing mice scheduled to receive diphtheria toxin, five of 10 mice had 1–5 seizures during baseline monitoring, while three of five DTr-expressing controls and one of seven DTr-negative controls had 3–10 and 5 seizures, respectively. The two control groups did not differ, so they were pooled for subsequent statistical analyses. Median seizure frequency did not differ between controls (*n* = 12 mice) and Vgat ablation mice (*n* = 10) during either the baseline week [Fig. 2*D*; Mann–Whitney RST with Bonferroni’s corrections (*α* set to 0.05/6 comparisons or <0.008)] or during the 5 d of diphtheria toxin treatment (*p* = 0.824 and 0.602, respectively).

### Ablation of hippocampal Vgat-expressing interneurons causes severe seizures

It takes ∼5 d for DTr-expressing cells to die following toxin exposure (Fink et al., 2014; Spampanato and Dudek, 2017). Correspondingly, quantification of seizure incidence in the first week after the 5 d diphtheria toxin treatment revealed dramatic seizure clusters in Vgat ablation animals relative to controls (Fig. 2*D*; *n* = 12 controls and 10 ablation; RST, *p* < 0.001). In addition, 6 of 10 Vgat ablation mice exhibited periods of persistent epileptiform activity, defined as an epileptiform EEG signal lasting longer than 5 min. Periods of persistent epileptiform activity lasted for up to 2 h in some ablation mice but were never observed in controls. All control animals survived the first week after ablation (Week 1), while one Vgat ablation animal died 3 d after the end of diphtheria toxin treatment during a seizure event.

EEG monitoring was continued for an additional 2–4 weeks to assess the persistence of seizure activity. During the second week after diphtheria toxin treatment, seizure activity declined precipitously in the Vgat ablation mice, and median seizures/day did not differ between groups (Fig. 2*D*; *n* = 12 controls and 9 ablation; RST; *p* < 0.913). One Vgat ablation mouse was removed during the second week due to an electrode failure. In the third week after treatment, an uptick in seizures in ablated mice produced a significant difference between groups (Fig. 2*D*; *n* = 10 controls and 8 ablation; RST; *p* = 0.002), although the effect was more modest, with only a single animal exceeding 10 seizures/day, while most animals had 0–3 seizures/day. The significant increase persisted into the fourth week, with more frequent seizures among ablation mice relative to controls (Fig. 2*D*; *n* = 9 control and 8 Vgat ablation mice; RST; *p* = 0.002).

### Seizure duration and behavior

Qualitative observations suggested that seizures were milder at first, followed by worsening severity and the onset of persistent epileptiform activity in some animals. To better understand the course of seizures in the animals, seizure duration and behavior were scored. Plotting behavioral seizure scores for Vgat ablation mice against seizure number revealed a slight dip in behavioral severity at the 5–10 seizure mark (Fig. 2*E*), followed by a relative plateauing with most subsequent seizures being convulsive (bilateral forelimb clonus and/or rearing and falling). Analysis of seizure duration was more striking, with initial seizures being relatively brief, followed by a dramatic increase in duration between seizure numbers 15–65 (Fig. 2*F*). This corresponds to the subset of animals that developed persistent epileptiform activity. Interestingly, after ∼65 seizures, duration decreased back to initial values.

### Baseline seizure incidence does not appear to confound responses to Vgat ablation

To query whether the occurrence of baselines seizures modulated responses to interneuron ablation, we conducted correlation analyses between pre- and postablation seizure incidence. Correlation analyses revealed no significant relationship between baseline seizure frequency and seizure frequency during any subsequent epochs (Spearman rank order correlations; diphtheria toxin, *R* = −0.114, *p* = 0.733; Week 1, *R* = −0.579, *p* = 0.074; Week 2, *R* = 0.242, *p* = 0.491; Week 3, *R* = 0.392, *p* = 0.321; Week 4, *R* = −0.173, *p* = 0.662). There was also no relationship between the presence or absence of baseline seizures and the occurrence of persistent epileptiform activity. Four of five mice without baseline seizures and two of five with baseline seizures developed persistent epileptiform activity (*z* test; *p* = 0.197).

### Diphtheria toxin treatment does not alter seizures in Vgat-FlpO+ mice not expressing the DTr

In Experiment 1, many Vgat-FlpO+ mice and their littermates exhibited baseline seizures, indicating that a subset of animals had pre-existing epilepsy (at least following EEG surgery). We were concerned, therefore, that these epileptic Vgat-FlpO+ mice might develop seizures after diphtheria toxin treatment due to nonspecific effects of the toxin. To test this possibility, four Vgat-FlpO+ mice received hippocampal injections of an AAV9-frt-mCherry reporter construct followed by EEG implantation and toxin treatment along the same timeline as animals in Experiment 1 (Table 1, Experiment 2). As these animals cannot express the DTr, they should not respond to diphtheria toxin exposure. Animals were monitored through the baseline week, the 5 d of diphtheria toxin treatment, and the week after treatment. During baseline monitoring, three of four mice exhibited spontaneous seizures; however, despite already being epileptic, toxin treatment had no effect on mean seizure frequency (baseline, 0.929 ± 0.343 seizures/day; diphtheria toxin, 0.650 ± 0.320 seizures/day; Week 1, 0.450 ± 0.126 seizures/day; one-way repeated measures ANOVA; *p* = 0.324). These findings indicate that nonspecific effects of diphtheria toxin treatment in Vgat-FlpO+ mice are unlikely to account for the dramatic spike in seizures observed in the ablated mice.

### Specificity, distribution, and efficacy of DTr expression

**Figure 3.**
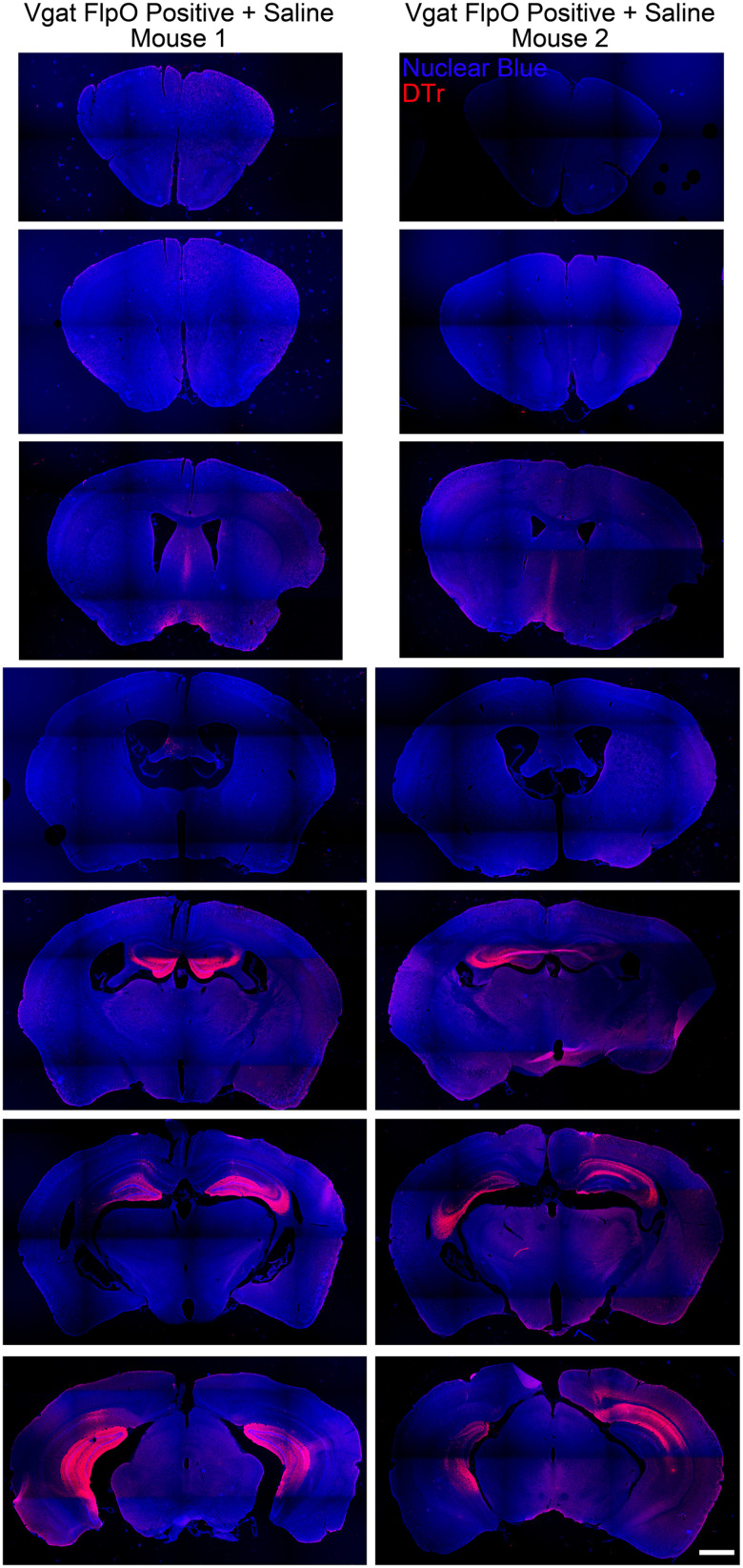
Dorsal–ventral series of coronal sections from two Vgat-FlpO–positive mice treated with saline. These mice express the DTr in the same pattern expected for Vgat ablation animals; however, since the mice were not treated with diphtheria toxin, the receptor is still present. The distribution of DTr expression is shown by immunostaining for the receptor (red). Strong expression is evident in the hippocampi of both animals. Scale bar, 1,000 µm. Additional details on the pattern of DTr expression are provided in Extended Data Figure 3-1.

To assess the potential extent of targeted interneurons, the pattern of DTr expression among the five Vgat-FlpO–positive mice treated with saline was examined through serial sections of each brain (Fig. 3). Robust labeling was restricted to the dentate gyrus and CA3 in all five animals, with lighter labeling in CA1 and subiculum. Subtle extrahippocampal labeling was evident in the fimbria, medial septum, and the diagonal band of Broca, likely reflecting axonal uptake of AAV9 from hippocampal-projecting GABAergic neurons. Two animals had subtle cortical labeling along the needle tract and one animal had subtle labeling in the geniculate nuclei and reticular thalamus. Semiquantitative regional scores for each animal are provided in Extended Data Figure 3-1.

10.1523/ENEURO.0317-23.2024.f3-1 Figure 3-1 Pattern of DTr expression in DTr-expressing mice treated with saline. Download Figure 3-1, DOCX file.

**Figure 4.**
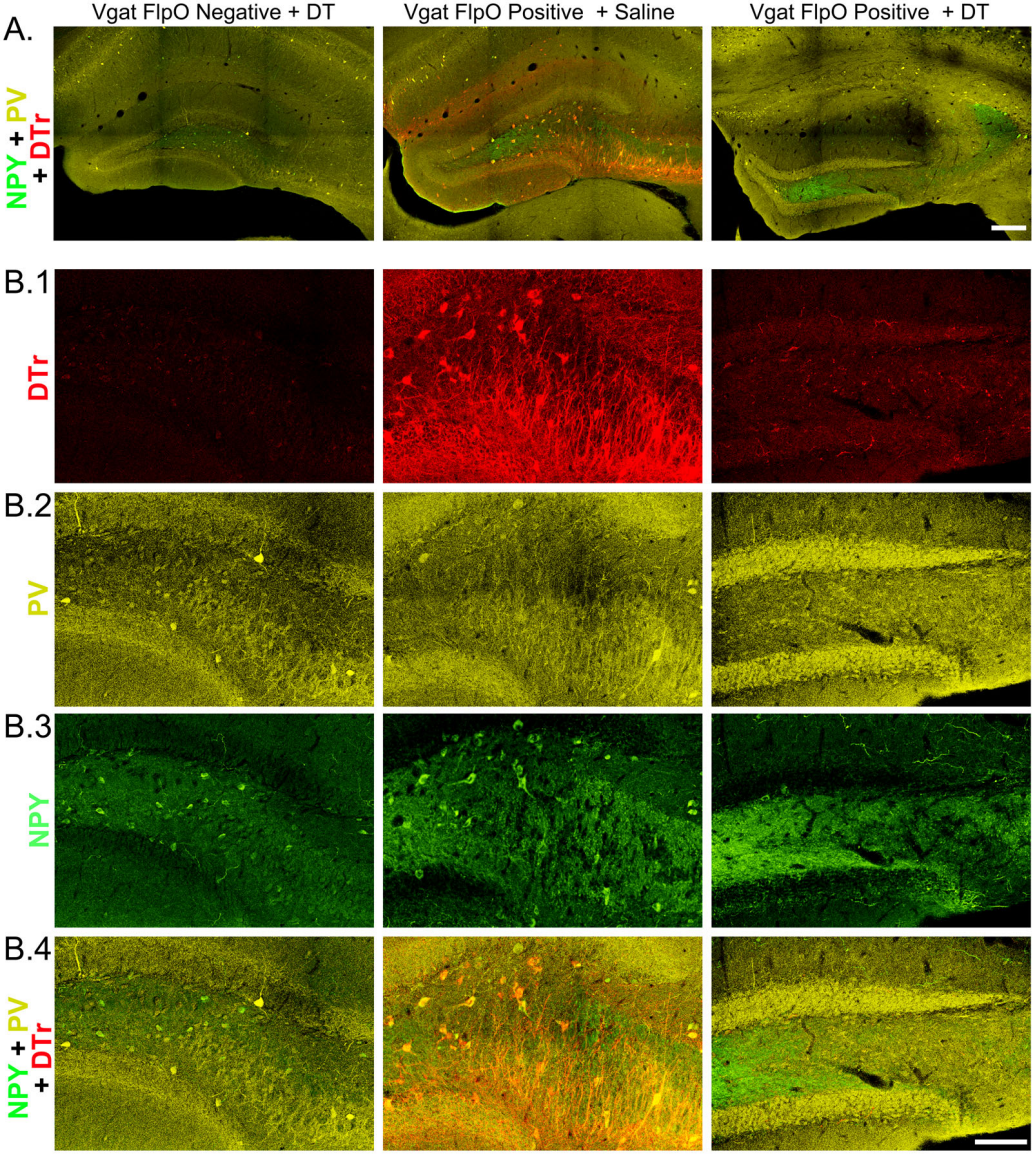
***A***, Confocal images of the hippocampal dentate gyrus showing DTr (red), parvalbumin (PV, yellow), and neuropeptide Y (NPY, green) immunostaining in control (Vgat-FlpO negative + DT, Vgat-FlpO positive + saline) and Vgat ablation mice (Vgat-FlpO positive + DT). Scale bar, 200 µm. ***B***, Higher-resolution images of the dentate gyri shown in ***A***. Scale bar, 100 µm. ***B.1***, DTr immunoreactivity is absent in the FlpO-negative animal (left), while robust labeling is evident in the Vgat-FlpO–positive mouse treated with saline (middle). DTr immunoreactivity is largely absent from the Vgat ablation animal (right) after the death of expressing neurons. ***B.2***, Small numbers of PV immunoreactive interneurons are evident in control mice (left, middle), while these neurons are absent following ablation (right). ***B.3***, NPY immunoreactive hilar interneurons are present in large numbers in control mice (left, middle), while NPY immunoreactive cell bodies are absent after ablation. Increased NPY labeling in the hilus of the Vgat ablation mouse reflects upregulation of this peptide in granule cell mossy fiber axons. ***B.4***, Merged channels show colocalization of DTr with PV and NPY immunoreactive interneurons in control mice (middle).

To directly assess ablation efficacy, brain sections were immunostained for DTr and the interneuron markers parvalbumin, neuropeptide Y (NPY) and somatostatin. DTr immunostaining confirmed that the protein was absent from Vgat-FlpO–negative mice, while Vgat-FlpO–positive mice treated with saline exhibited robust immunoreactivity in the dentate gyrus and CA3 (Fig. 4*A*). DTr immunoreactivity was overtly reduced in Vgat ablation mice, although rare DTr-immunoreactive cells and processes could be found on close analysis (Fig. 4*B.1*). Coimmunostaining with DTr and the interneuron markers parvalbumin (Fig. 4*B.2*) and NPY (Fig. 4*B.3*) revealed extensive colocalization in Vgat-FlpO–positive mice treated with saline and reductions in hippocampal interneurons in Vgat ablation mice. DTr immunoreactivity also colocalized with somatostatin (data not shown). Interestingly, NPY immunoreactivity was increased in the granule cell mossy fiber pathway (hilus and stratum lucidum) in some Vgat ablation mice, consistent with previous studies demonstrating production of NPY by granule cells in epilepsy models (Nadler et al., 2007).

**Figure 5.**
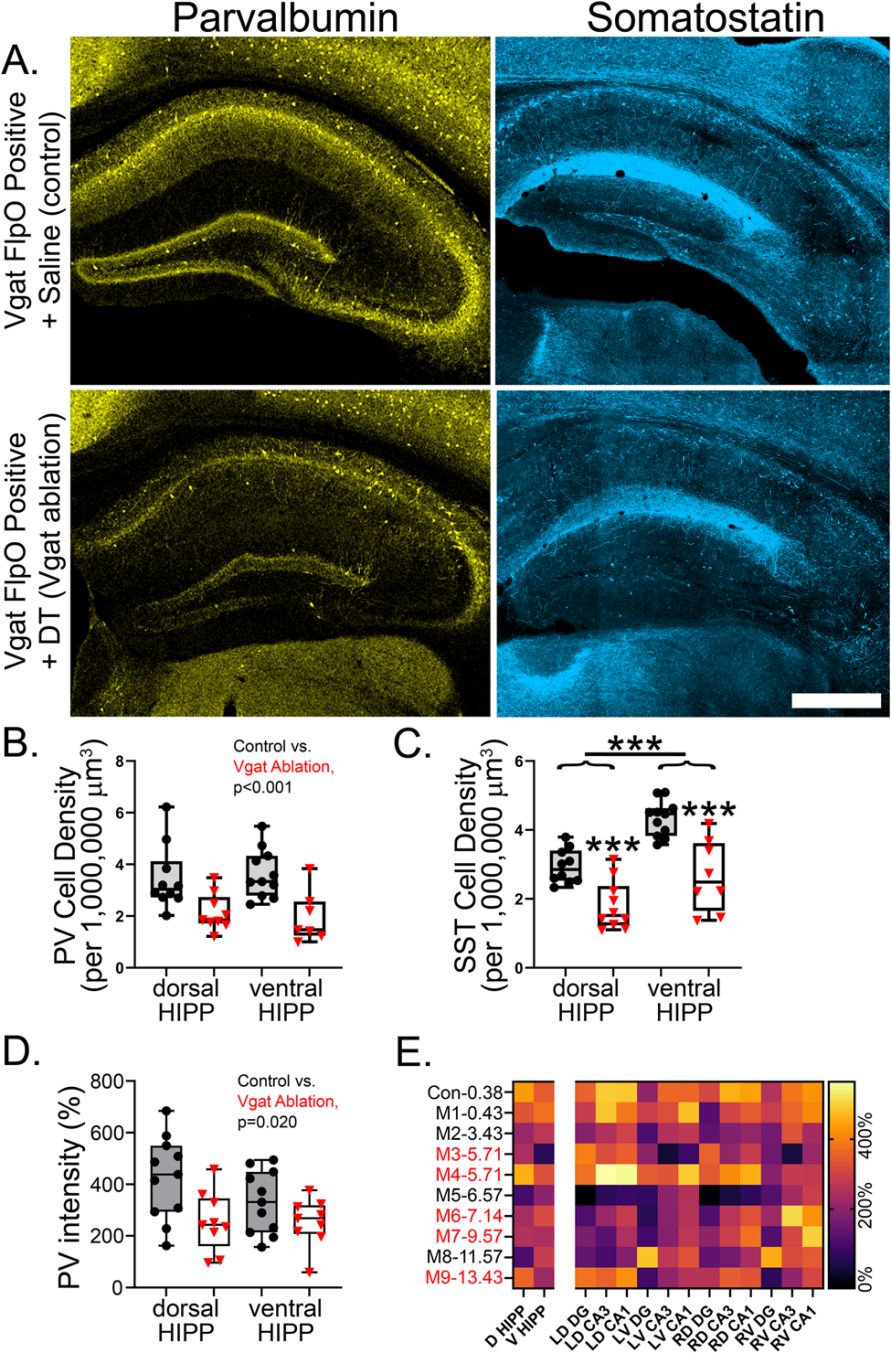
***A***, Parvalbumin (PV) and somatostatin (SST) immunostaining in the hippocampi of control and Vgat ablation mice. Reduced immunoreactivity is evident after ablation. Scale bar, 500 µm. ***B***, Ablation significantly reduced the density of hippocampal PV neurons (*p* < 0.001, main effect of ablation). ***C***, Ablation significantly reduced the density of hippocampal somatostatin (SST) neurons in both dorsal and ventral hippocampus. Cell counts also revealed an effect of level, with more SST cells present in ventral hippocampus. ***D***, The intensity of PV immunostaining in dorsal and ventral hippocampus (HIPP), expressed as the percentage over PV signal intensity in corpus callosum, was significantly reduced after ablation. ***E***, Heat map of PV immunostaining intensity (right *y*-axis, 0–600% over corpus callosum) for each Vgat ablation mouse (M1–M9) plotted against average data for controls (Con, top row). Numbers on the left *y*-axis give average seizures/day for each mouse. Red text indicates animals that developed persistent epileptiform activity. Columns give data for dorsal (D) and ventral (V) hippocampus (the same data plotted in ***B***) and individual measures from left (L) and right (R) dentate gyrus (DG), CA3 and CA1. ***p < 0.001.

To quantify ablation efficacy, the density of parvalbumin and somatostatin immunoreactive neurons (Fig. 5*A*) was determined in sections from dorsal and ventral hippocampus in each animal. The density of parvalbumin immunoreactive neurons was reduced to 62.5 and 52.5% of control levels for the dorsal and ventral hippocampus, respectively [Fig. 5*B*; two-way repeated measures ANOVA with treatment (control or ablation) and level (dorsal/ventral) as factors; main effect of treatment; *p* < 0.001]. There was no significant effect of level (*p* = 0.920) nor was there a significant interaction (*p* = 0.510). Within the dentate gyrus, parvalbumin neuron density was reduced to 52.5% of control levels in the dorsal and 57.3% in the ventral hippocampus (control × dorsal, 1.065 ± 0.120 parvalbumin cells/1,000,000 µm^3^; control × ventral, 0.968 ± 0.120; ablation × dorsal, 0.559 ± 0.143; ablation × ventral, 0.555 ± 0.143; *p* = 0.003). A similar reduction in somatostatin interneurons was found, with density reduced to 63.9% of control values in the dorsal hippocampus and 60.2% in the ventral hippocampus (Fig. 5*C*; two-way repeated measures ANOVA; *p* < 0.001). A significant difference between the dorsal and ventral hippocampus was also observed, with somatostatin neuron density being greater in the ventral hippocampus (*p* < 0.001). Within the dentate gyrus, ablation was particularly effective, with somatostatin neuron density reduced to 27.4 and 33.3% of control values in dorsal and ventral hippocampus, respectively (control × dorsal, 1.920 ± 0.268 somatostatin cells/1,000,000 µm^3^; control × ventral, 2.646 ± 0.268; ablation × dorsal, 0.527 ± 0.282; ablation × ventral, 0.881 ± 0.282; *p* < 0.001).

Parvalbumin neurons are relatively few in number in the intact hippocampus; however, they field an extensive axonal arbor, with each parvalbumin neuron contacting thousands of cells (Hu et al., 2014). Small numbers of surviving parvalbumin cells, therefore, can have large effects. As an additional measure of ablation efficacy, therefore, we quantified parvalbumin staining intensity for the entire hippocampus for each animal. Importantly, this approach captures the large axonal plexus of parvalbumin neurons. Ablation reduced the intensity of hippocampal parvalbumin immunoreactivity to 60.2% of control levels in the dorsal and 76.7% in the ventral hippocampus [Fig. 5*D*; two-way ANOVA with treatment (control or ablation) and level (dorsal/ventral) as factors; main effect of group (*p* < 0.020) with no significant effect of level (*p* = 0.140) or interactions (*p* = 0.153)]. To further parse the pattern of reduced parvalbumin immunoreactivity, staining intensity was collected for the dentate gyrus, CA3 subregion, and CA1 subregion, producing a total of 12 ROIs (3 regions × dorsal/ventral × left/right) for each mouse. A more granular analysis of the 12 ROIs revealed a diversity of patterns in Vgat ablation mice, with no clear relationship between parvalbumin immunoreactivity in a specific ROI and seizure frequency (Fig. 5*E*). The lack of a relationship is interpreted cautiously, however, as each ablation pattern was unique and thus not suited for well-powered statistical analysis.

### Perisomatic inhibitory terminals

**Figure 6.**
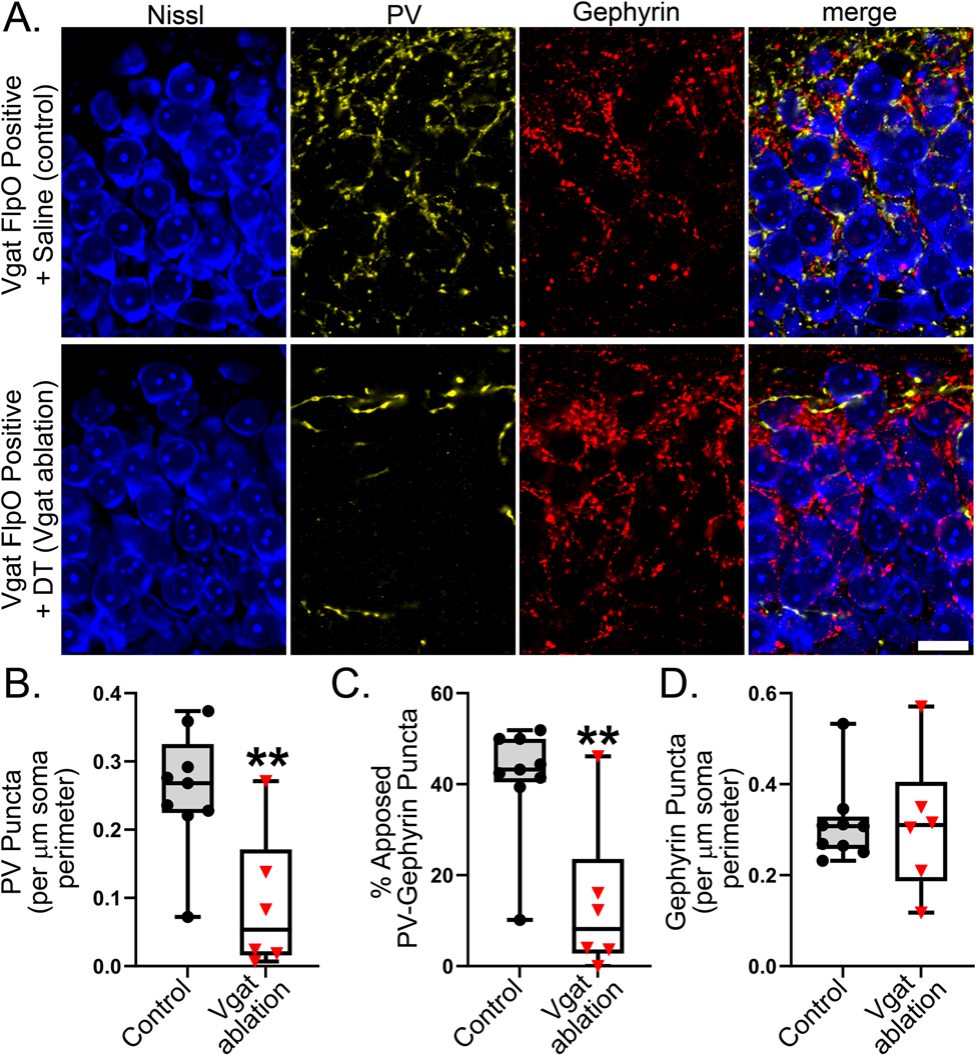
***A***, Parvalbumin (PV, yellow) and gephyrin (red) immunostaining shows perisomatic inhibitory inputs to granule cell somas (counterstained blue). Scale bar, 10 µm. ***B***, ***C***, The density of parvalbumin puncta surrounding granule cell somas, and the percentage of parvalbumin puncta apposed to gephyrin puncta (presumptive PV→granule cell synapses) is significantly reduced in Vgat ablation mice relative to controls. ***D***, The density of gephyrin puncta, marking a postsynaptic component of inhibitory synapses, is preserved despite the dramatic loss of parvalbumin puncta. **p < 0.01.

A key subset of parvalbumin-expressing interneurons inhibits excitatory neurons by forming “baskets” of perisomatic inputs around excitatory neuron somas. To assess changes in perisomatic input after interneuron ablation, parvalbumin immunostaining, to reveal presynaptic terminals, was combined with immunostaining for gephyrin, a postsynaptic scaffolding protein specific to inhibitory synapses (Fig. 6*A*). The number of parvalbumin puncta per length of soma perimeter was significantly reduced in Vgat ablation mice relative to controls (Fig. 6*B*; *n* = 9 control and 6 Vgat ablation mice; *p* = 0.005; *t* test). Correspondingly, the percentage of parvalbumin puncta apposed to gephyrin puncta (presumptive PV→granule cell synapses) was also reduced (Fig. 6*C*; *p* = 0.003; *t* test). Surprisingly, however, the density of gephyrin puncta did not differ significantly between controls and Vgat ablation mice (Fig. 6*D*; *p* = 0.906; RST). Taken together, findings support the interpretation that ablation produces a dramatic reduction in granule cell perisomatic input from parvalbumin interneurons—which does not recover—but also suggest that this input may have been replaced by another (parvalbumin immunonegative) interneuron class.

### Transient silencing of Vgat neurons causes ISs and seizures

**Figure 7.**
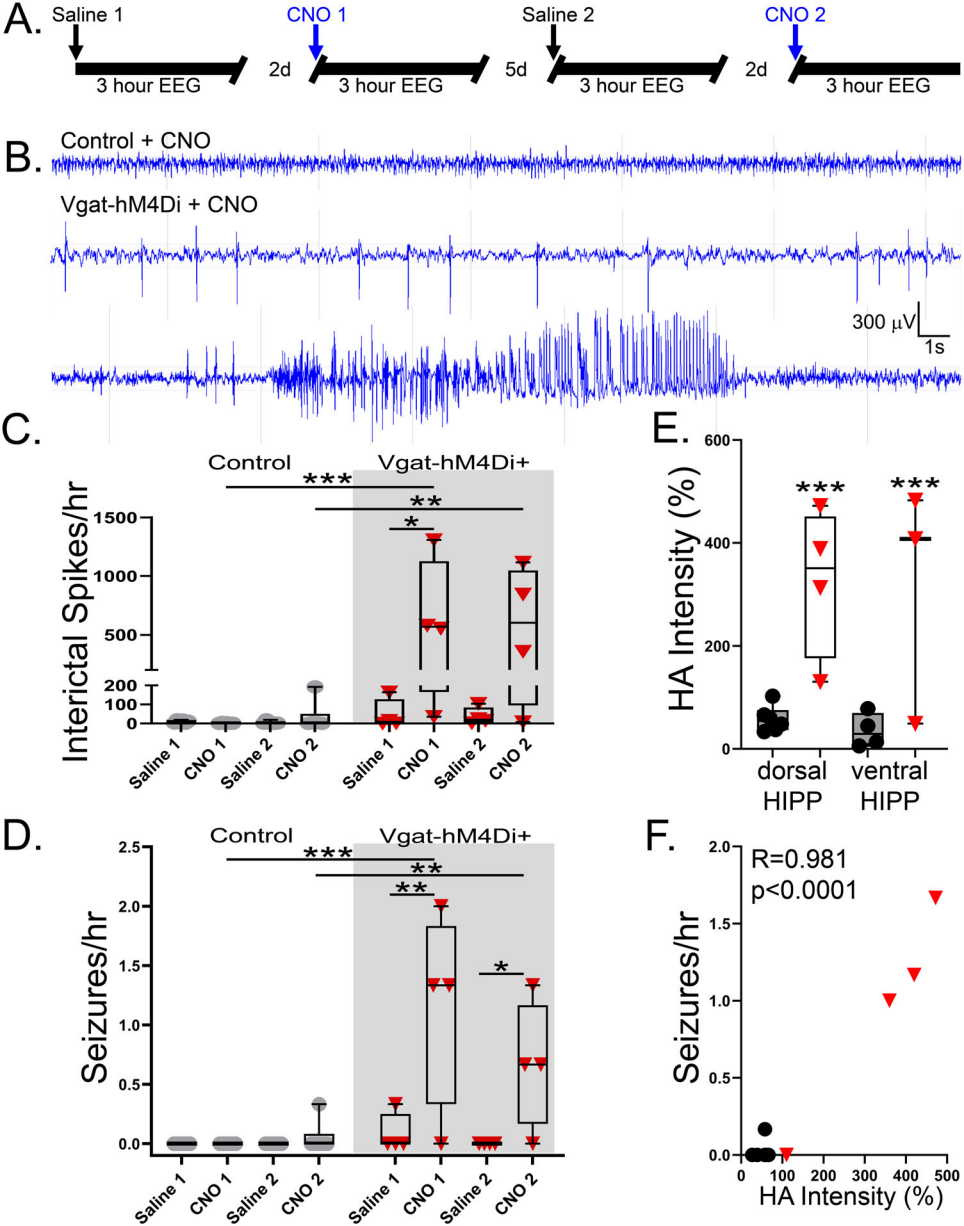
***A***, Interneuron silencing protocol with saline (control) and CNO treatments interspersed by 2 and 5 d drug wash-out periods. ***B***, EEG responses to CNO treatment from a control mouse, showing a normal EEG (top trace), and Vgat-hM4D_i_+ mice, showing ISs (middle trace), and a seizure (bottom trace) after CNO treatment. ***C***, Box and whisker plots (interquartile range with minimum to maximum and individual animals plotted) show the dramatic increase in the frequency of ISs in Vgat-hM4D_i_+ mice treated with CNO (red triangles) relative to control animals (gray circles). Note the break in the *y*-axis. ***D***, Box and whisker plots showing that CNO treatment significantly increased seizure occurrence in Vgat-hM4D_i_+ mice but had no effect in controls. ***E***, The intensity of HA immunostaining in dorsal and ventral hippocampus (HIPP), expressed as the percentage over HA signal intensity in corpus callosum, was significantly increased in Vgat-hM4D_i_+ mice. ***F***, Average seizure frequency following CNO treatment was significantly correlated with the intensity of HA immunostaining. *p < 0.05; **p < 0.01; ***p < 0.001.

Evidence in the present study of sprouting from surviving interneurons complicates interpretation of ablation experiments. Diphtheria toxin-mediated ablation of interneurons also has the potential to cause nonspecific changes, such as inflammation, that could exacerbate seizures. Chemogenetic neuronal silencing offers an alternate strategy to explore interneuron function without injuring the target cells. We therefore utilized a parallel AAV vector and approach to introduce the inhibitory chemogenetic (DREADD) silencing receptor hM4D_i_ into Vgat-FlpO+ (Vgat-hM4D_i_+) and control mice (Table 1, Experiment 3). In addition, since the hM4D_i_ ligand CNO has a half-life of only a few hours, we were able to use a repeated measures design, with each animal receiving two control and two CNO treatments over a period of 2 weeks (Fig. 7*A*; saline 1, CNO1, saline 2, CNO2).

Silencing Vgat+ interneurons with CNO produced a dramatic 10-fold increase in ISs during the 3 h time window following injection (Fig. 7*B*). Within Vgat-hM4D_i_+ mice, spikes following saline treatments averaged 45 ± 40 and 36 ± 23/h, jumping to 619 ± 262 and 581 ± 247/h after CNO. Spikes appeared rapidly, with frequency equaling or exceeding five ISs/minute an average of 9.2 ± 0.77 min after CNO injection of Vgat-hM4D_i_+ mice. Statistical analyses revealed a significant interaction between genotype (Vgat-hM4D_i_+ vs control) and drug treatment (saline vs CNO) among the groups (Fig. 7*C*; two-way repeated measures ANOVA on ranked data; *p* = 0.014). Post hoc tests showed a significant increase in IS following CNO treatment of Vgat-hM4D_i_+ mice relative to CNO-treated controls for both the first (Bonferroni’s *t* test; *p* < 0.001) and second (*p* = 0.006) rounds. Spike frequency was also significantly increased following the first round of CNO treatment of Vgat-hM4D_i_+ mice relative to the preceding saline treatment in the same animals (Bonferroni’s *t* test; *p* < 0.043). CNO treatment had no significant effects on saline controls.

CNO treatment induced seizures in 75% of Vgat-hM4D_i_+ mice (Fig. 7*D*). Full details on the latency, duration, and severity of each seizure are provided in Extended Data Figure 7-1. In each case, seizures appeared after the onset of IS, with a mean latency of 16.4 ± 3.2 min. Statistical analyses revealed a significant interaction between genotype (Vgat-hM4D_i_+ vs control) and drug treatment (saline vs CNO) in the mice (two-way repeated measures ANOVA on ranked data, *p* = 0.004). Post hoc analyses confirmed that CNO treatment of Vgat-hM4D_i_+ mice increased seizure frequency relative to the corresponding saline treatment of the same animals (drug within Vgat-hM4D_i_+, CNO1 vs saline 1, Bonferroni’s *t* test; *p* = 0.008; CNO2 vs saline 2, *p* = 0.002). CNO had no effect in control mice (CNO1 vs saline 1, *p* = 1.000; CNO2 vs saline 2, *p* = 1.000). The response of Vgat-hM4D_i_+ mice to CNO also differed from the control animal response to CNO (Vgat-hM4D_i_+ CNO1 vs control CNO1, *p* < 0.001; Vgat-hM4D_i_+ CNO2 vs control CNO2, *p* = 0.005). Vgat-hM4Di+ mice had a total of 21 seizures during CNO treatment periods. Forty-five percent of seizures were electrographic only (no overt behavior), 35% were limbic (freezing/facial automatisms), and 20% were convulsive. Seizures ranged in duration from 13 to 172 s (mean 40.2 ± 7.8 s). Findings demonstrate that abrupt silencing of hippocampal Vgat+ interneurons can lead to the rapid occurrence of seizures; however, responses were milder than Vgat neuron ablation. Animals only had a few seizures during the observation period, and no animals developed persistent epileptiform activity.

10.1523/ENEURO.0317-23.2024.f7-1 Figure 7-1 Metrics for each seizure following transient silencing of Vgat neurons. Seizures occurring in Vgat-hM4Di+ mice during CNO treatment periods are highlighted. Download Figure 7-1, DOCX file.

### Specificity and spread of hM4D_i_ receptor expression

**Figure 8.**
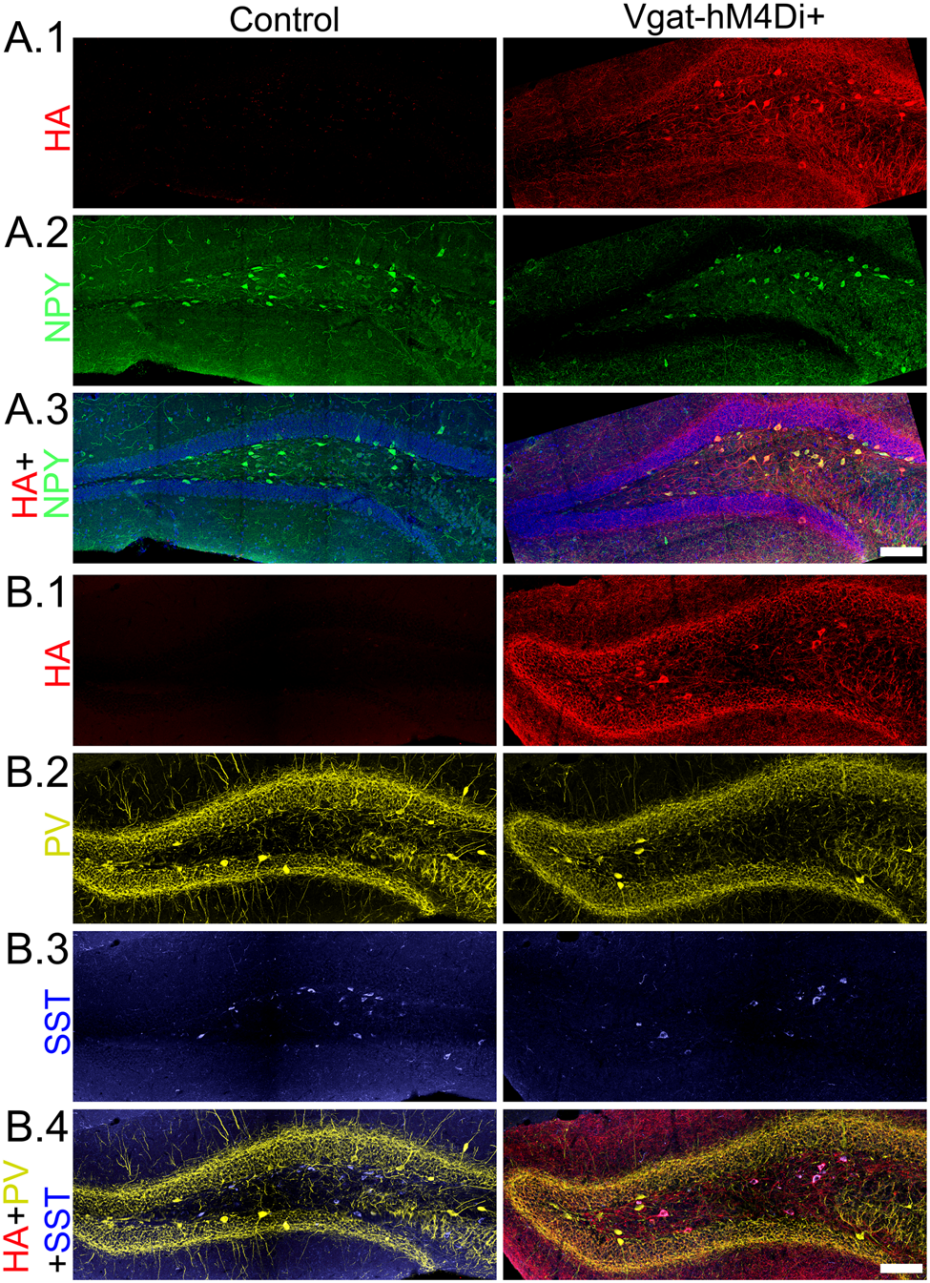
***A***, Confocal images of the hippocampal dentate gyrus showing labeling for nuclear blue (blue), HA-tagged hM4D_i_+ receptors (***A.1***, red), and NPY (***A.2***, green) in control (Vgat-FlpO negative+ AAV9-hM4D_i_/HA) and Vgat-hM4D_i_+ mice (Vgat-FlpO–positive+ AAV9-hM4D_i_/HA). ***B***, Confocal images from control and Vgat-hM4D_i_+ mice showing colocalization of HA (***B.1***, red) with parvalbumin (***B.2***, PV, yellow) and somatostatin (***B.3***, SST, blue). Scale bar, 100 µm.

Histological studies were conducted in all animals to confirm hM4D_i_/HA expression in hippocampal interneurons. HA immunoreactivity was significantly increased in both the dorsal and ventral hippocampus relative to controls [Fig. 7*E*; two-way ANOVA on ranked data with treatment group and level (dorsal/ventral) as factors; main effect of group (*p* < 0.001) with no significant effect of level (*p* = 0.439) or interactions (*p* = 0.287); *p* < 0.001 for “group within level” for the dorsal and ventral hippocampus, Holm–Sidak]. In addition, the one Vgat-hM4D_i_+ animal that did not develop seizures after CNO had the lowest level of HA immunoreactivity, suggesting that the absence of a response reflects poor expression of the receptor. Indeed, the intensity of HA immunoreactivity was strongly correlated with seizure frequency during CNO treatment periods (Fig. 7*F*; Pearson’s correlation; *R* = 0.981; *p* < 0.0001). Coimmunostaining for HA and NPY, parvalbumin, or somatostatin confirmed excellent colocalization between HA and the interneuron markers (Fig. 8). No evidence of significant nonspecific labeling of excitatory principal cells was evident.

### Vgat silencing increases absolute gamma and reduces relative theta and alpha power

**Figure 9.**
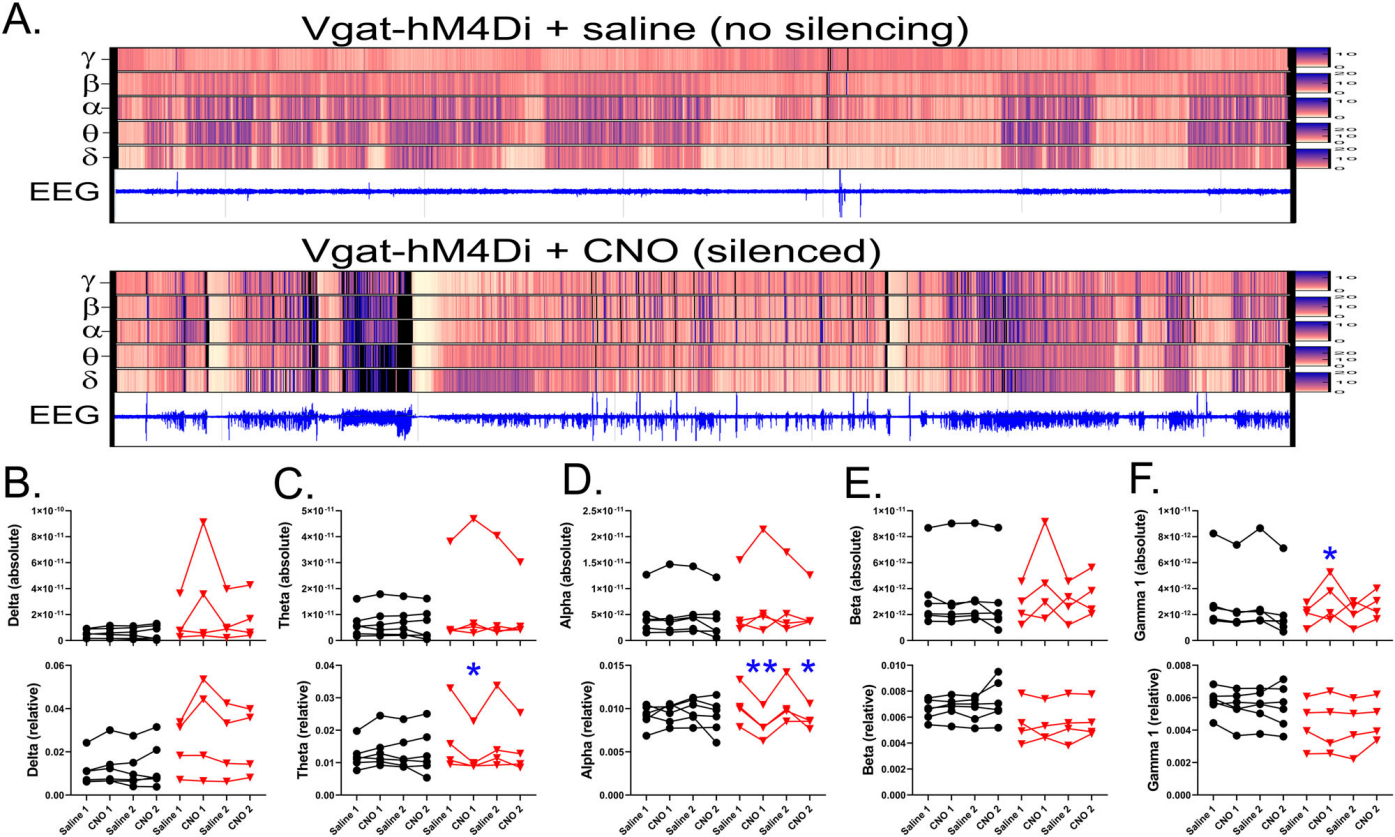
***A***, Heat map of EEG power within delta, theta, alpha, beta, and gamma frequency bands from a single Vgat-hM4D_i_+ mouse during the 3 h period following saline (top) or CNO (bottom) treatment. The scale for each band is shown on the right. EEG recordings for the same period are in blue. ***B–F***, Absolute (top) and relative (bottom) power for delta (***B***), theta (***C***), alpha (***D***), beta (***E***), and gamma (***F***) bands. Each line shows the data from a single animal during the four treatment periods (saline 1, CNO 1, saline 2, CNO 2). Control animals are depicted in black and Vgat-hM4D_i_+ animals in red. *p < 0.05. **p < 0.01.

Interneurons are implicated in driving EEG oscillations, so we queried whether these were altered by interneuron silencing. Silencing significantly increased absolute gamma power [Fig. 9; two-way repeated measures ANOVA with animal and treatment (saline or CNO) as factors, square root transformation; interaction *p* = 0.004, CNO1 vs saline 1 within Vgat-hM4D_i_+ mice, *p* = 0.047]. A significant interaction was also detected for the beta band (*p* = 0.049); however, this was not significant by Bonferroni’s post-test (CNO1 vs saline 1 within Vgat-hM4D_i_+ mice, *p* = 0.105). No differences in absolute EEG power were evident in the delta (group, *p* = 0.171; treatment, *p* = 0.223; interaction, *p* = 0.279), theta (group, *p* = 0.487; treatment, *p* = 0.267; interaction, *p* = 0.764), or alpha (group, *p* = 0.573; treatment, *p* = 0.260; interaction, *p* = 0.525) bands.

Relative power was decreased by interneuron silencing in the theta (Fig. 9; interaction, *p* = 0.009; CNO1 vs saline 1 within Vgat-hM4D_i_+ mice, *p* = 0.021) and alpha (interaction, *p* = 0.010; CNO1 vs saline 1 within Vgat-hM4D_i_+ mice, *p* = 0.004; CNO2 vs saline 2 within Vgat-hM4D_i_+ mice, *p* = 0.038) bands. No differences in relative EEG power were evident in the delta (group, *p* = 0.164; treatment, *p* = 0.186; interaction, *p* = 0.960), beta (group, *p* = 0.138; treatment, *p* = 0.153; interaction, *p* = 0.732) or gamma (group, *p* = 0.187; treatment, *p* = 0.395; interaction, *p* = 0.345) bands.

## Discussion

The pathophysiological significance of interneuron loss in epilepsy remains controversial, with evidence suggesting it is a key driver of the disease (Bumanglag and Sloviter, 2018), while other studies suggest it is one change among many that contribute to epileptogenesis (Dudek and Staley, 2012; Hester and Danzer, 2013). Prior studies have utilized focal unilateral targeted ablation and silencing strategies to isolate the impact of interneuron disruption, producing modest and sometimes transient periods of seizures. For the present study, we extend this work by conducting expansive, bilateral dorsal–ventral ablation and silencing of interneurons in what is considered to be one of the most epileptogenic regions of the brain, the hippocampal dentate gyrus (Toyoda et al., 2013; Buckmaster et al., 2022). Ablation led to the occurrence of dozens of seizures and periods of persistent epileptiform activity over a timeframe of ∼1 week. Thereafter, however, seizure incidence declined precipitously, and persistent epileptiform activity did not recur. Transient interneuron silencing, in contrast, led to the occurrence of ISs and small numbers of seizures, but not persistent epileptiform activity. Findings provide further evidence that interneuron loss is sufficient to induce seizures and support the straightforward prediction that greater interneuron loss will drive more severe seizures. The striking improvement in the animal's condition in the second week after ablation, however, suggests either that acute seizures are driven by factors other than interneuron loss per se and that the brain possess substantial capacity to restore homeostasis in the face extensive interneuron loss—or some combination of the two.

### Extensive interneuron ablation produces severe seizures

For the present study, we examined the impact of an expansive interneuron ablation from bilateral dorsal and ventral hippocampus. We attempted this more expansive ablation in part because of prior studies showing relatively modest effects of more limited ablations. Specifically, unilateral hippocampal CA1 ablation of GABAergic neurons using a DTr expression strategy in Gad2-Cre mice produced up to six seizure/day (Spampanato and Dudek, 2017), with seizures resolving after the first week in most animals. Similarly, unilateral silencing of parvalbumin neurons in subiculum by AAV-mediated tetanus toxin expression produced a similar result, with an average of 1.5 seizures/week, declining slightly over a 6 week observation period (Drexel et al., 2017). Permanent unilateral silencing of somatostatin interneurons in subiculum produced a more modest effect, with ∼40% of mice developing seizures at a frequency of 1.2/month (Drexel et al., 2022). Chemical ablation strategies have also produced modest effects. Bilateral targeting of interneurons with substance P-saporin toxin conjugates, for example, caused brief seizures in the first week, followed by the appearance of spontaneous seizures months later (Chun et al., 2019). Here, we show that bilateral ablation of interneurons from the hippocampus is sufficient to reproduce a phenotype more akin to systemic treatments with GABA antagonists like pentylenetetrazol (PTZ) and bicuculline, generating robust seizures and persistent epileptiform activity.

### Significance of the transient seizure spike and recovery following ablation

The most surprising aspect of the present study is the relative recovery of animals following abrupt bilateral interneuron loss and dozens of seizures. Unlike seizure induction with GABA antagonists, which are quickly metabolized, ablated interneurons are permanently eliminated. So, what accounts for the dramatic reduction in seizures after the first week? A possible explanation is that the seizure clusters are mediated by acute injury and/or inflammatory changes rather than a direct effect of interneuron loss. Diphtheria toxin treatment takes about a week to kill DTr-expressing cells (Spampanato and Dudek, 2017), so the period of peak seizure incidence immediately follows cell death. Interneuron ablation can reasonably be viewed as a brain injury, and acute seizure in humans is common during the 1 week period following such injuries. These acute seizures are not considered to be epileptic, often resolving entirely after the first week (Mauritz et al., 2022). Consistent with the interpretation that the seizure clusters are injury induced, seizures following diphtheria toxin-mediated ablation of interneurons in CA1 followed an almost identical timeline, appearing in the week after toxin treatment and resolving in all but one animal thereafter (Spampanato and Dudek, 2017). In contrast, tetanus toxin silencing of interneurons in the subiculum—which did not kill the cells—produced a more steady-state induction of seizures without an obvious peak (Drexel et al., 2017, 2022). The comparatively modest effect of chemogenetic silencing of an identical population of interneurons in the present study also suggests that cell killing may have add-on effects. On the other hand, diphtheria toxin-mediated ablation of newborn hippocampal granule cells has been shown to decrease seizure incidence in the pilocarpine model of epilepsy (Hosford et al., 2016, 2017), demonstrating that this mode of cell killing is not necessarily ictogenic.

An alternative explanation for the resolution of the seizure clusters after ablation is the induction of homeostatic mechanisms to re-establish excitatory/inhibitory balance. In epilepsy models, surviving interneurons can receive increased excitatory drive (Halabisky et al., 2010; Hunt et al., 2011) and undergo sprouting to restore lost input (W. Zhang et al., 2009; Thind et al., 2010; Christenson Wick et al., 2017). Given the direct targeting of interneurons here, one is tempted to postulate compensation by surviving inhibitory cells. Indeed, while anatomical analyses revealed a striking reduction in perisomatic parvalbumin innervation of granule cells, the density of postsynaptic gephyrin immunoreactive puncta was not reduced. Several possibilities could explain this observation. Parvalbumin input may have been replaced by another interneuron population, although examination of somatostatin and NPY immunoreactivity did not reveal sprouting of either subtype in the granule cell body layer (data not shown). Alternatively, surviving parvalbumin neurons might reinnervate the cells, but with immunohistochemically undetectable levels of parvalbumin in the sprouted axons. An additional possibility is that gephyrin could persist at postsynaptic sites despite loss of presynaptic terminals, so putative synapses may be nonfunctional. Electrophysiological and anatomical studies are needed to confirm functionality and identify the source of any new input. In addition to interneurons, potential homeostatic mechanisms are expansive and could involve many cell types (Simons et al., 2023). An interesting example that was observed here is the increased expression of anticonvulsant NPY by excitatory granule cells (Fig. 4*B.3*). A variety of homeostatic and circuit changes to reduce excitatory and increase inhibitory drive, therefore, could be occurring during the week after ablation to reduce seizure incidence.

An additional intriguing possibility revolves around an expanding literature suggesting that interneurons themselves could initiate seizures (Levesque et al., 2019; Magloire et al., 2019; Raimondo and Dulla, 2019; Rich et al., 2020). Although seemingly paradoxical, evidence that interneurons become more active prior to seizure onset (Toyoda et al., 2015) has led to the hypothesis that interneurons might be acting to synchronize the activity of excitatory cells, making seizures likely once inhibition subsides. Such a mechanism would require sufficient numbers of surviving interneurons, as well as coordinated interplay among the different interneuron subtypes, which include interneurons that inhibit excitatory cells (net inhibitory) and interneurons that inhibit other inhibitory neurons (net excitatory). It is conceivable, therefore, that ablating too many interneurons or that simultaneous ablation of neurons with opposing net effects on network activity could limit the ability of a circuit to generate seizures. Additional studies are needed to explore these possibilities.

### Is interneuron loss sufficient to cause epilepsy?

The present findings are consistent with previous studies indicating that interneuron loss alone can cause epilepsy (Drexel et al., 2017, 2022; Spampanato and Dudek, 2017; Chun et al., 2019), with some key caveats. Most notably, baseline seizures were observed in half of the mice undergoing subsequent interneuron ablation. Baseline seizures occurred after a 2 week surgery recovery period, moving them out of the range of acute symptomatic (nonepileptic) seizures. Behavioral seizures were not observed in the mice prior to EEG surgery during routine handling, although infrequent behavioral seizures are hard to detect without 24/7 monitoring, and seizures without behavioral manifestations cannot be detected at all without EEG. Assuming seizures were absent prior to surgery, the events might reflect a post-traumatic epilepsy from surgical procedures, as has been observed in prior studies (Jiang et al., 2015; Lybrand et al., 2021). Seizure occurrence in controls, however, was more frequent than in our prior studies (Castro et al., 2012; Rowley et al., 2017; LaSarge et al., 2021). Spontaneous epilepsy-sensitizing mutations have been characterized in other mouse lines (Yang et al., 2003), and a similar Vgat-Cre line has been found to exhibit reduced Vgat mRNA and protein, with enhanced kindling epileptogenesis, suggesting that creation of the cre-line disrupted the endogenous Vgat gene (Straub et al., 2020). Regardless, the presence of seizures prior to ablation precludes establishing whether ablation causes epilepsy, as the animals were already epileptic. That said, ablation worsened epilepsy in the mice. A second key caveat is that 60% of the ablation mice developed persistent epileptiform activity, which might induce proepileptogenic changes in addition to interneuron loss. Finally, we note that the cortical electrodes used in the present study may not always detect seizures occurring in deeper brain structures, so it is possible that future studies with depth electrodes might produce differing findings.

### Transient versus permanent interneuron silencing

For the present study, transient interneuron silencing with DREADDs was compared with interneuron ablation. Similar to prior work in which permanent interneuron silencing with tetanus toxin was compared with transient silencing with DREADDs (Drexel et al., 2017), transient silencing had a comparatively mild impact on seizure activity. For the present study, DTr and hM4D_i_ genes were carried by AAV vectors with identical backbones, injected at identical concentrations at identical coordinates. A trivial explanation for the limited efficacy of DREADDs is that hM4D_i_-mediated cell silencing is less effective than tetanus toxin or cell ablation. Alternatively, it is possible that the duration of silencing is important. Spontaneous seizures appeared an average of 10 d after AAV-tetanus toxin injection (Drexel et al., 2017), and persistent epileptiform activity events did not appear until after ∼20 brief seizures following ablation (Fig. 2*F*). The observations raise the possibility that chronic interneuron silencing initiates secondary mechanisms that exacerbate ictogenesis.

### Brain rhythms

Brain activity is characterized by rhythmic patterns of oscillatory activity. Parvalbumin interneurons are implicated in driving gamma rhythms (Bartos et al., 2007; Hu et al., 2014), which are often associated with cognitive processes (Kahana, 2006; Mably and Colgin, 2018). Changes in multiple frequency bands have been observed in animals and patients with epilepsy (Lenck-Santini and Sakkaki, 2022). Here, we took advantage of the neuronal silencing approach to conduct within-animal analyses, revealing an increase in absolute gamma power. Increased gamma is unexpected, given prior findings that suppressing parvalbumin neurons reduces gamma, while activation increases gamma (Cardin et al., 2009; Sohal et al., 2009). The interneuron silencing associated with the Vgat-FlpO mouse line likely produced complex changes, including alterations in other interneuron populations, including subtypes that inhibit other interneurons (Tsodyks et al., 1997). However, such paradoxical findings may be better understood at the systems level by considering cortical network models. Inhibition stabilization is a common motif in cortical networks (Antoine et al., 2019; Sanzeni et al., 2020); the loss of inhibitory input may precipitate network destabilization leading to compensatory activity (Lovelace et al., 2020). For example, interneurons not expressing the hM4D_i_ receptor might increase activity and contribute to increased gamma (Strüber et al., 2022). Alternatively, it has also been proposed that increased spontaneous gamma may not reflect interneuron-driven gamma synchrony per se but rather an overall increase in circuit activity (Sohal and Rubenstein, 2019; Guyon et al., 2021). In addition to gamma changes, we observed reduced relative power in the theta and alpha bands, meaning that these frequencies are smaller components of total EEG power with interneurons silenced. Theta is associated with cognition, while alpha correlates with quiet restfulness in humans (Lenck-Santini and Sakkaki, 2022; Quigley, 2022). Reduced power in these bands would be hypothesized to affect cognitive function and states of consciousness (H. Zhang et al., 2018), and future studies could use our approach for behavioral testing.

### Conclusions

Here, we challenged the brain by inducing widespread loss of hippocampal interneurons. Ablation produced transient seizure clusters followed by a persistent but more modest increase in seizures. Findings support a critical role for interneuron loss in epileptogenesis but also demonstrate a surprising capacity for the brain to maintain relative homeostasis in the face of a widespread disinhibition event.

## Synthesis

Reviewing Editor: William Stacey, University of Michigan Decisions are customarily a result of the Reviewing Editor and the peer reviewers coming together and discussing their recommendations until a consensus is reached. When revisions are invited, a fact-based synthesis statement explaining their decision and outlining what is needed to prepare a revision will be listed below. The following reviewer(s) agreed to reveal their identity: Gilles Huberfeld, Vincent Magloire.

The reviewers and I have discussed your responses and agree that most of the concerns have been answers sufficiently. We have one further concern that should be addressed prior to publication.

The reviewers do not believe the data shown as evidence of status epilepticus is indisputably compatible with that term. Given this uncertainty, and the questionable applicability of those patterns to human status epilepticus, we request that you change the terminology. We agreed that this pattern of prolonged/recurrent epileptic discharges is clearly severely pathologic, but it should be called something less categorical instead of status epilepticus. You could discuss the relationship and similarities to status, but we did not feel it appropriate to claim this was true status.

These terminology changes should be reflected in the title, abstract, and text.

## Author Response

We thank the editors and reviewers for their helpful suggestions and critiques. Please see our point-by-point responses below (AR) and highlighted revisions in the manuscript text.

Synthesis of Reviews:

Synthesis Statement for Author (Required):

The reviewers and I have discussed your responses and agree that most of the concerns have been answers sufficiently. We have one further concern that should be addressed prior to publication.

The reviewers do not believe the data shown as evidence of status epilepticus is indisputably compatible with that term. Given this uncertainty, and the questionable applicability of those patterns to human status epilepticus, we request that you change the terminology. We agreed that this pattern of prolonged/recurrent epileptic discharges is clearly severely pathologic, but it should be called something less categorical instead of status epilepticus. You could discuss the relationship and similarities to status, but we did not feel it appropriate to claim this was true status.

These terminology changes should be reflected in the title, abstract, and text.

AR: The term "status epilepticus" has been replaced with "persistent epileptiform activity" through the title, abstract, text and figures.
