# Supplementary material for: Transient Seizure Clusters and Epileptiform Activity Following Widespread Bilateral Hippocampal Interneuron Ablation
Source: eNeuro. 2024 Apr 16;11(4):ENEURO.0317-23.2024. doi: 10.1523/ENEURO.0317-23.2024 (PMC11036118; doi:10.1523/ENEURO.0317-23.2024)
Supplement: Figure 3-1 — Pattern of DTr expression in DTr-expressing mice treated with saline. Download Figure 3-1, DOCX file. [file eneuro-11-ENEURO.0317-23.2024-s001.docx]

**Figure 3-1:** Pattern of DTr expression in DTr-expressing mice treated with saline

|  | DG | CA3 | CA1 | Sub | Fimbria | MS | DBB | CTX | GN | RTN |
| --- | --- | --- | --- | --- | --- | --- | --- | --- | --- | --- |
|  | L/R | L/R | L/R | L/R |  |  |  |  |  |  |
| VM290101 | 2.4 / 0.1 | 2.5 / 0 | 1.6 / 0.4 | 0 / 0 | 0.6 | 1 | 0 | 0.1 | 0.3 | 0.2 |
| VM260401 | 2.9 / 2.0 | 3.0 / 2.5 | 0.7 / 0.8 | 0 / 0.1 | 0.4 | 0.7 | 1.3 | 0 | 0 | 0 |
| VM280407 | 2.0 / 0.6 | 2.7 / 0.3 | 0 / 1.4 | 0.3 / 0.9 | 0.1 | 0.6 | 0.7 | 0.1 | 0 | 0 |
| VM290401 | 0.8 / 2.1 | 1.4 / 1.9 | 0.3 / 0.3 | 0.3 / 1.0 | 0.2 | 0.8 | 0.9 | 0 | 0 | 0 |
| VM290402 | 3.0 / 3.0 | 3.0 / 3.0 | 0 / 0.1 | 0.3 / 0.3 | 0.7 | 0.8 | 0.9 | 0 | 0 | 0 |

L/R, left/right hemisphere. DG, dentate gyrus. Sub, subiculum. MS, medial septum. DBB, diagonal band of Broca. CTX, cortex. GN, geniculate nuclei. RTN, Reticular thalamus.
