# Supplementary material for: Transient Seizure Clusters and Epileptiform Activity Following Widespread Bilateral Hippocampal Interneuron Ablation
Source: eNeuro. 2024 Apr 16;11(4):ENEURO.0317-23.2024. doi: 10.1523/ENEURO.0317-23.2024 (PMC11036118; doi:10.1523/ENEURO.0317-23.2024)
Supplement: Figure 7-1 — Metrics for each seizure following transient silencing of Vgat neurons. Seizures occurring in Vgat-hM4Di+ mice during CNO treatment periods are highlighted. Download Figure 7-1, DOCX file. [file eneuro-11-ENEURO.0317-23.2024-s002.docx]

**Figure 7-1:** Metrics for each seizure following transient silencing of Vgat neurons. Seizures occurring in Vgat-hM4Di+ mice during CNO treatment periods are highlighted.

| **Animal** | **Group** | **Treatment** | **Readout** | **Seizure 1** | **Seizure 2** | **Seizure 3** | **Seizure 4** | **Seizure 5** | **Seizure 6** |
| --- | --- | --- | --- | --- | --- | --- | --- | --- | --- |
| VM330102 | control | ALL 4 | n/a | NONE |  |  |  |  |  |
|  |  |  |  |  |  |  |  |  |  |
| VM330103 | control | ALL 4 | n/a | NONE |  |  |  |  |  |
|  |  |  |  |  |  |  |  |  |  |
| VM330104 | control | ALL 4 | n/a | NONE |  |  |  |  |  |
|  |  |  |  |  |  |  |  |  |  |
| VM330107 | control | ALL 4 | n/a | NONE |  |  |  |  |  |
|  |  |  |  |  |  |  |  |  |  |
| VM330108 | control | Saline 1 | n/a | NONE |  |  |  |  |  |
| VM330108 | control | CNO1 | n/a | NONE |  |  |  |  |  |
| VM330108 | control | Saline 2 | n/a | NONE |  |  |  |  |  |
| VM330108 | control | CNO2 | Latency | 78m 36s |  |  |  |  |  |
| VM330108 | control | CNO2 | Duration | 14.65 |  |  |  |  |  |
| VM330108 | control | CNO2 | Racine | 4 |  |  |  |  |  |
|  |  |  |  |  |  |  |  |  |  |
| VM330111 | control | ALL 4 | n/a | NONE |  |  |  |  |  |
|  |  |  |  |  |  |  |  |  |  |
| VM330101 (+) | Vgat-hM4Di+ | Saline1 | Latency | 113m 47s |  |  |  |  |  |
| VM330101 (+) | Vgat-hM4Di+ | Saline1 | Duration | 37.1 |  |  |  |  |  |
| VM330101 (+) | Vgat-hM4Di+ | Saline1 | Racine | 4 |  |  |  |  |  |
| VM330101 (+) | Vgat-hM4Di+ | CNO1 | Latency | 13m 24s | 17m 38s | 59m 54s | 67m 32s | 79m 51s | 119m 3s |
| VM330101 (+) | Vgat-hM4Di+ | CNO1 | Duration | 21.98 s | 78.65 s | 36.65 s | 46.62 s | 38.35 s | 28.23 s |
| VM330101 (+) | Vgat-hM4Di+ | CNO1 | Racine | 0 | 4 | not visible | 1 | 5 | 0 |
| VM330101 (+) | Vgat-hM4Di+ | Saline2 | n/a | NONE |  |  |  |  |  |
| VM330101 (+) | Vgat-hM4Di+ | CNO2 | Latency | 11m 46s | 19m 41s | 52m 31s | 78m 12s |  |  |
| VM330101 (+) | Vgat-hM4Di+ | CNO2 | Duration | 13.37 | 17 | 24.87 | 20.6 |  |  |
| VM330101 (+) | Vgat-hM4Di+ | CNO2 | Racine | 0 | 0 | 1 | 0 |  |  |
|  |  |  |  |  |  |  |  |  |  |
| VM330105 (+) | Vgat-hM4Di+ | Saline1 | n/a | NONE |  |  |  |  |  |
| VM330105 (+) | Vgat-hM4Di+ | CNO1 | Latency | 15m 32s | 31m 13s | 44m 46s | 119m 43s |  |  |
| VM330105 (+) | Vgat-hM4Di+ | CNO1 | Duration | 21.77 | 87.6 | 171.6 | 25.58 |  |  |
| VM330105 (+) | Vgat-hM4Di+ | CNO1 | Racine | 1 | 5 | 5 | 1 |  |  |
| VM330105 (+) | Vgat-hM4Di+ | Saline2 | n/a | NONE |  |  |  |  |  |
| VM330105 (+) | Vgat-hM4Di+ | CNO2 | Latency | 30m 54s |  |  |  |  |  |
| VM330105 (+) | Vgat-hM4Di+ | CNO2 | Duration | 30.39 |  |  |  |  |  |
| VM330105 (+) | Vgat-hM4Di+ | CNO2 | Racine | 1 |  |  |  |  |  |
|  |  |  |  |  |  |  |  |  |  |
| VM330106 (+) | Vgat-hM4Di+ | Saline1 | n/a | NONE |  |  |  |  |  |
| VM330106 (+) | Vgat-hM4Di+ | CNO1 | Latency | 9m 11s | 33m 14s | 74m 50s | 107m 46s |  |  |
| VM330106 (+) | Vgat-hM4Di+ | CNO1 | Duration | 51.92 | 27.67 | 15.43 | 42.72 |  |  |
| VM330106 (+) | Vgat-hM4Di+ | CNO1 | Racine | 0 | 0 | 0 | 0 |  |  |
| VM330106 (+) | Vgat-hM4Di+ | Saline2 | n/a | NONE |  |  |  |  |  |
| VM330106 (+) | Vgat-hM4Di+ | CNO2 | Latency | 21m 28s | 61m 47s |  |  |  |  |
| VM330106 (+) | Vgat-hM4Di+ | CNO2 | Duration | 20.4 | 21.81 |  |  |  |  |
| VM330106 (+) | Vgat-hM4Di+ | CNO2 | Racine | 1 | 2 |  |  |  |  |
|  |  |  |  |  |  |  |  |  |  |
| VM330109 (+) | Vgat-hM4Di+ | ALL 4 | n/a | NONE |  |  |  |  |  |
